# TIMP1 Derived from Mesenchymal Stem Cells Promotes Bladder Cancer Progression by Regulating the Formation of VDIMs through the RAP1 Pathway

**DOI:** 10.7150/ijbs.130720

**Published:** 2026-03-17

**Authors:** Pan Li, Enguang Yang, Xinyu Zhang, Yibo Shi, Chaohu Chen, Guangrui Fan, Yuhan Wang, Hanzhang Wang, Nan Liu, Hong Zhou, Junqiang Tian, Liang Cheng, Zhilong Dong, Zhiping Wang

**Affiliations:** 1 Department of Urology, the Second Hospital & Clinical Medical School, Lanzhou University, Lanzhou, China.; 2 Department of Urological Oncology, Chongqing University Cancer Hospital, Chongqing, China.; 3 The Legorreta Cancer Center at Brown University, Department of Pathology and Laboratory Medicine, The Warren Albert Medical School of Brown University, Brown University Health, Providence, RI, USA.

**Keywords:** Bladder cancer, Tissue inhibitor of metalloproteinases 1, RAS-related protein 1, Vesicles derived from the inner mitochondrial membrane.

## Abstract

The pro-tumor function of mesenchymal stem cells (MSCs) in bladder cancer (BC) is not fully elucidated. This study integrates clinical cohorts, organoid models, and patient-derived xenografts (PDX) to dissect MSCs-derived TIMP1 as a key driver of BC progression. Using multiplex fluorescent immunohistochemistry and enzyme-linked immunosorbent assays, we found that elevated infiltration level of MSCs in BC tissues and TIMP1 levels in tissues/urine correlated with advanced tumor-stage, lymphovascular invasion, and reduced recurrence-free survival time, with MSCs infiltration positively associated with TIMP1 expression. Single-cell data analysis and mass spectrometry revealed TIMP1 as the predominant cytokine secreted by MSCs. Mechanistically, MSC-derived TIMP1 binds to ADAM10 to inhibit its extracellular shedding, thereby stabilizing cMet phosphorylation and activating the RAP1 signaling axis. Functional studies revealed that TIMP1 enhances intracellular Ca^2+^ levels and VDAC1 expression through the RAP1 pathway, promoting the formation of vesicles derived from the inner mitochondrial membrane (VDIMs) to regulate mitochondrial quality control. Crucially, the TIMP1 inhibitor FXR agonist 3 suppressed MSCs-driven BC proliferation *in vitro* and attenuated tumor growth in PDX models by disrupting the cMet-RAP1 signaling pathway without systemic toxicity. Our findings propose targeting the MSCs-TIMP1-RAP1 axis as a novel therapeutic strategy for BC.

## Introduction

Bladder cancer (BC) is the tenth most common malignancy worldwide, with its high recurrence rate and therapeutic resistance posing significant challenges 1. Recent research suggests that mesenchymal stem cells (MSCs) in the tumor microenvironment (TME), acting as stromal cells with self-renewal and multipotent differentiation capabilities, affect tumor cells through the secretion of proteins, lipids, RNAs, metabolites, and other mechanisms 2, 3. Our prior study indicated that the metabolites produced by MSCs can regulate the progression of BC 4. Meanwhile, the protein products secreted by MSCs may stimulate the growth of BC. However, what kinds of components and mechanisms are involved remain ambiguous.

Tissue inhibitor of metalloproteinases 1 (TIMP1) is a glycoprotein that inhibits metalloproteinases and was initially regarded as a protective factor against tumor proliferation 5. Subsequently, TIMP1 expression was markedly elevated in the majority of tumor tissues and the blood of cancer patients, correlating with a poor prognosis and perhaps facilitating tumor growth 6, 7. As a cytokine, it influences TME remodeling and modulates tumor cell proliferation by interacting with the quadruple transmembrane proteins CD63 and CD82 8, 9. Previous studies on tumors primarily focused on the influence of TIMP1 produced by tumor cells, neglecting the contribution of TIMP1 derived from other significant stromal cells within the TME. Therefore, the secretion level of TIMP1 by MSCs in the BC microenvironment is still unknown, and the correlation between its expression level and the clinicopathological features as well as tumor development in BC patients remains ambiguous. Moreover, several extracellular cytokines primarily modulate cellular processes via membrane-associated pathways, including the G protein-coupled receptor system, enzyme-linked receptor pathway, and ion channel receptors, among others 10-12. Nonetheless, it is uncertain whether TIMP1 inside the TME may modulate alterations in tumor biological activity via mechanisms other than its interaction with quadruple transmembrane proteins.

Mitochondrial homeostasis is essential for maintaining mitochondrial activity inside cells. Cells utilize many quality control systems, such as mitochondrial biogenesis 13, dynamics 14, protein homeostasis 15, mitophagy 16, and mitochondrial-derived vesicles (MDVs) 17, to preserve the structural and functional integrity of mitochondria. Our prior research has demonstrated that MSCs inside the BC microenvironment can facilitate the advancement of BC via modulating mitochondrial autophagy 4. Recent research has revealed that damage to mitochondrial cristae leads to alterations in cytosolic Ca^2+^ levels, resulting in the detachment of impaired inner mitochondrial membrane fragments that form vesicle-like structures known as vesicles derived from the inner mitochondrial membrane (VDIMs), which are subsequently internalized by adjacent lysosomes 18. This reveals a novel mechanism of mitochondrial quality control, defined as MitoTracker(+)/TOM20(-) and significantly different from previously found MDVs regarding content, size, and formation process 18, 19. The formation of MDVs is highly dependent on dynamin-related protein 1, with the biogenesis of a subset of MDVs also mediated by sorting nexin 9; moreover, their generation requires inductive stimulation by amino acids. In contrast, the formation of VDIMs is independent of dynamin-related protein 1, mitochondrial Rho GTPase 1 and sorting nexin 9, which does not need exogenous induction and can be detected under cellular resting state 20-22. The possibility of MSCs controlling mitochondrial homeostasis through the modulation of mitochondrial VDIMs release in tumor cells remains uncertain.

The present study assessed the relationships between the infiltration level of MSCs, the expression of TIMP1, and the adverse clinical features of BC utilizing clinical tissue, urine, and single-cell omics data. Simultaneously, it was investigated how MSCs-secreted TIMP1 regulates the biological behavior alterations of BC and how it affects tumor cells' formation of VDIMs to support mitochondrial quality control. This study also utilized a TIMP1 inhibitor and a patient-derived xenograft (PDX) model from BC patients to investigate the reduction of the stimulatory effect of MSCs on tumor progression by TIMP1 secretion inhibition, therefore establishing a foundation for novel therapeutic approaches for BC.

## Methods

### Patient samples and publicly available data

This study retrospectively collected postoperative tissue samples from 56 patients diagnosed with BC after excluding patients with a previous history of tumors. Additionally, preoperative morning urine samples were collected from 42 BC patients, as well as morning urine samples from 8 non-cancer patients. Transcriptome data from the BLCA cohort were obtained from the UCSC Xena database (https://xena.ucsc.edu), and MSCs scores were computed via the x_Cell method 23. The GSE222315 dataset, which includes single-cell sequencing data from 4 normal bladder tissue samples and 9 BC samples, was sourced from the Gene Expression Omnibus collection 24.

### Single-cell data processing

Single-cell data was processed using the Omicverse package 25. The specific workflow is as follows: Scrublet was used to identify doublet cells, and cells with nUMI less than 500, detected genes less than 250, and a mitochondrial gene count ratio greater than 15% were filtered out. Subsequently, the data were normalized sequentially, high-variability genes were identified, dimensionality was decreased, and clustering was performed. Manual and automatic annotations were performed based on a marker dictionary of MSCs-specific genes and genes specifically expressed in different cell types within the tumor tissue 26.

### Multiplex immunohistochemical (mIHC)

Tissue microarrays were prepared from patients within the past year for mIHC using the TSA plus Fluorescent Triplex/Quadruplex Staining Kit (Servicebio Cat# G1236). The experimental procedures were followed according to the manufacturer's instructions. HRP-conjugated secondary antibodies bound to CD73 (Proteintech Cat# 12231-1-AP, RRID: AB_2154081; 1:250), CD90 (Proteintech Cat# 27178-1-AP, RRID:AB_3085933; 1:500), and CD105 (Proteintech Cat# 10862-1-AP, RRID:AB_2098906; 1:1000) were amplified using different TSA dyes. The Strata Quest Tissue Flow Quantitative Analysis Platform (Tissue Gnostics, Austria) was utilized to identify and quantify triple-positive cells.

### Immunohistochemistry (IHC)

Paraffin-embedded 4 μm slices were dewaxed and rehydrated. After antigen retrieval in citrate buffer, 3% H_2_O_2_ suppressed endogenous peroxidase. After intervention with TIMP1 and EPAC1 antibodies (Proteintech Cat# 16644-1-AP, RRID:AB_2878292; 1:250 and Proteintech Cat# 12572-1-AP, RRID:AB_11232407; 1:250, respectively), the sections were treated with enzyme-labeled secondary antibodies. We visualized with DAB and counterstained with hematoxylin. Tissue Gnostics Panoramic Tissue Cell Scanning System (Tissue Gnostics, Austria) images were analyzed using Image J for average optical density (AOD).

### Cell culture

The MSCs were sourced from the American Type Culture Collection and grown under standard circumstances using the designated medium supplied by the supplier. The multilineage differentiation potential of MSCs was validated via adipogenic, osteogenic and chondrogenic induction assays **(Fig. S1A-C)**. The UMUC-3 (RRID:CVCL_1783) and J82 (RRID:CVCL_0359) cell lines were procured from the Shanghai Cell Bank of the Chinese Academy of Sciences and regularly maintained in RPMI 1640 medium supplemented with 10% fetal bovine serum and 1% antibiotic solution. The HEK-293T cell (RRID:CVCL_KS57) line, sourced from the Shanghai Cell Bank of the Chinese Academy of Sciences, was consistently grown in high-glucose DMEM supplemented with 10% FBS and 1% antibiotic solution.

### Enzyme-linked immunosorbent assay (ELISA)

Human and mice urine were centrifuged and stored at -80 °C. Supernatants from cell cultures were harvested after 24 hours of incubation and subsequently subjected to centrifugation. TIMP1 concentrations were measured with a commercial human TIMP1 ELISA kit (Elabscience Cat# DY970) and a mice TIMP1 ELISA kit (Jonlnbio Cat# JL12876). Samples were diluted 1:5 with sample diluent and placed in wells, then incubated for 1.5 hours following the manufacturer's guidance. After extracting the well liquid, biotinylated antibody and HRP enzyme conjugate working solution were added. After 8 minutes using TMB substrate chromogenic solution, a stop solution stopped the reaction. The absorbance was measured at 450 nm.

### Analysis of conditioned medium (CM) mass spectrometry

T75 cell culture flasks were used to cultivate MSCs. The media was changed to 10 mL of high-glucose DMEM with 10% FBS after the cell density reached 80%. The cells were then cultivated for a further 24 hours. To get rid of cell debris, the collected MSCs-CM was centrifuged at 3000×g for 10 minutes at 4 °C. High-performance liquid chromatography-mass spectrometry analysis was then performed on the cell supernatant.

### Cell counting kit-8 (CCK-8) assay

Upon reaching the intervention time, the original media in each well was removed, and 100 μL of cell culture medium containing 10% CCK-8 solution (Yeasen Cat# 40203ES80) was introduced into each well. The culture plate was thereafter positioned in a cell incubator and incubated for 2 hours in darkness. The absorbance of each well was measured at 450 nm using a microplate reader.

### Cell cloning

Remove the culture supernatant from the well plate and rinse once with PBS buffer. Introduce 1 ml of 4% paraformaldehyde into each well, incubate at ambient temperature in darkness for 30 minutes, and subsequently wash once with PBS buffer. Add 1 mL of crystal violet working solution to each well and incubate the cells for 30 minutes. Following the disposal of the crystal violet solution, the cells were rinsed three times with PBS. Subsequently, the picture is generated following the drying of the well plate.

### Lentivirus packaging

Competent bacteria (DH5α, Jikai Company) were thawed on ice and combined with 1μL of plasmid solution (TIMP1-shRNA1: 5′-GAAGTCAACCAGACCACCTTA-3′; shRNA2: 5′-ACAGACGGCCTTCTGCAATTC-3′; shRNA3: 5′-GCACAGTGTTTCCCTGTTTAT-3′; Miaoling Biology). Following a 30-minute incubation on ice, cells were subjected to heat shock at 42 °C for 90 seconds and subsequently recovered in SOC medium at 37 °C for 40 minutes. Transformed bacteria were cultured on LB agar supplemented with antibiotics, and individual colonies were proliferated for plasmid extraction with a commercial kit (Tiangen Cat# DP123). HEK-293T cells were transfected with pLVX, psPAX2, and pMD2.G plasmids at a ratio of 4:2.3:1.5 μg with PEI reagent. Viral supernatants were harvested at 24 and 48 hours post-transfection, filtered using 0.45 μm membranes, and preserved at -80 °C. MSCs were transduced with lentivirus at a multiplicity of infection of 10, utilizing 8 μg/mL of polybrene. Puromycin selection was administered for 5 days, and the surviving cells were confirmed using western blot.

### Western blot

Proteins were extracted utilizing RIPA buffer including protease and phosphatase inhibitors, quantified by BCA assay, and resolved on 10% SDS-PAGE gels. Following transfer to PVDF membranes, blots were subjected to blocking with 5% non-fat milk and incubated overnight at 4°C with primary antibodies: EPAC1 (Proteintech Cat# 16644-1-AP, RRID:AB_2878292; 1:500), RASGRP2 (Proteintech Cat# 30189-1-AP, RRID:AB_3086257; 1:1000), RAP1A (Proteintech Cat# 68125-1-Ig, RRID:AB_2923653; 1:1000), ADAM10 (MedChemExpress Cat# HY-P83547, RRID:AB_3105656; 1:500), TUBULIN (Proteintech Cat# 66200-1-Ig, RRID:AB_2722562; 1:2000), Ph-cMet (MedChemExpress Cat# HY-P80803, RRID:AB_3102198; 1:1000), cMet (MedChemExpress Cat# HY-P80219, RRID:AB_3102357; 1:1000), VDAC1 (Proteintech Cat# 10866-1-AP, RRID:AB_2257153; 1:1000), TIMP1 (Proteintech Cat# 16644-1-AP, RRID:AB_2878292; 1:1,000), and GAPDH (Proteintech Cat# 60004-1-Ig, RRID:AB_2107436; 1:3000). IRDye-labeled secondary antibodies (LI-COR Biosciences Cat# 926-32210, RRID:AB_621842; 1:20000) were employed for fluorescence detection using the Two-color infrared laser imaging system (America, LI-COR).

### GST pulldown

The RAP1 Pulldown activation test kit (NewEast Cat# 81401) was utilized to evaluate RAP1 GTPase activity. The UMUC-3 and J82 cells were subjected to intervention with MSCs-CM, ESI-09 (10 µM), TIMP1 (12.5ng/mL), or their combinations for a duration of 24 hours. Cells were lysed in ice-cold 1× Assay/Lysis buffer supplemented with 1 mM PMSF. Clarified lysates were treated with anti-active RAP1 antibody and Protein A/G agarose beads for one hour at 4 °C. The beads were rinsed thrice with lysis buffer, and the bound proteins were eluted in 2× Laemmli buffer at 95°C for 5 minutes. Eluates were separated using 15% SDS-PAGE gels, transferred to PVDF membranes, and analyzed using anti-RAP1 antibodies.

### Immunofluorescence (IF)

Cells were fixed using 4% paraformaldehyde for 15 minutes at room temperature, thereafter washed with PBS, and permeabilized with 0.1% Triton X-100 (Beyotime Cat# P0096). Following blocking (Beyotime Cat# P0260), samples were incubated overnight at 4℃ with anti-His (Proteintech Cat# 66005-1-Ig, RRID:AB_11232599, 1:1,000) and anti-TIMP1 (Proteintech Cat# 16644-1-AP, RRID:AB_2878292; 1:500) primary antibodies. Secondary antibodies (Proteintech Cat# AF594-goat anti-rabbit, AF647-goat anti-mouse, 1:1000) and Actin-Tracker Green (1:100) were administered for 1 hour (room temperature, in darkness), followed by Hoechst (Solarbio Cat# C0031) nuclear staining for 10 minutes. Each process incorporated three washes with PBST. Imaging was conducted with a confocal microscope (Zeiss LSM880) equipped with suitable filters.

### Quantitative polymerase chain reaction (qPCR)

Total RNA was extracted utilizing RNA lysis reagent (Vazyme Cat# R701), followed by isopropanol precipitation and ethanol washes. Genomic DNA was removed using 4×gDNA Wiper Mix (42 °C for 2 minutes), followed by cDNA synthesis with 5×HiScript III qRT SuperMix (37 °C for 15 minutes; 85 °C for 5 seconds). qPCR was performed in 8 μL reactions comprising TB Green Premix Ex Taq II (4 μL), primers (0.5 μL each), and cDNA template (1 μL), with the following cycling parameters: 95 °C for 30 seconds; 40 cycles of 95 °C for 5 seconds and 60 °C for 30 seconds, succeeded by melt curve analysis. Primers were produced by Qingke Biotech with the following sequences:

GAPDH-Forward: 5′-ACAACTTTGGTATCGTGGAAGG-3′; GAPDH-Reverse: 5′-GCCATCACGCCACAGTTTC-3′. ADAM10-Forward: 5′-ATGGGAGGTCAGTATGGGAATC-3′; ADAM10-Reverse: 5′-ACTGCTCTTTTGGCACGCT-3′.

### Detection of Ca^2+^

Cells were dissociated with 0.25% trypsin, neutralized with serum-free media, and subjected to three washes with Ca²⁺/Mg²⁺-free HBSS. Fluo-4/AM stock solution was prepared with anhydrous DMSO, and then diluted to a 4 μM working solution using calcium-free HBSS solution. After resuspension in 500 μL of Fluo-4/AM working solution (4 μM, Yeasen Biotechnology Cat# 40704ES50), the cell suspension was incubated at 37 °C for 45 minutes under light-protected conditions. The residual extracellular probe was eliminated with three further rounds of Ca²⁺/Mg²⁺-free HBSS washing (1200×g, 3 minutes). Before analysis, cells were filtered through a 100-μm nylon mesh to achieve single-cell suspensions, thereafter examined using a Beckman CytoFLEX flow cytometer utilizing 488 nm excitation.

### Identification of VDIMs

Mitochondria were stained with 200 nM MitoTracker™ Red CMXRos (Thermo Fisher Scientific, Cat# M7512) in serum-free medium, followed by incubation at 37 °C for 30 minutes under light-protected conditions. Cells were fixed with 4% paraformaldehyde, permeabilized with 0.1% Triton X-100, then blocked using an immunostaining blocking solution. Primary antibodies targeting TOM20 (Proteintech Cat# 11802-1-AP, RRID:AB_2207530; 1:200) and LAMP1 (Proteintech Cat# 21997-1-AP, RRID:AB_2878966; 1:100) were incubated overnight at 4 °C, subsequently followed by a 1-hour incubation at room temperature with CoraLite488-conjugated goat anti-rabbit IgG (Proteintech Cat# SA00013-2, RRID:AB_2797132, 1:1000) and Alexa Fluor 647-conjugated goat anti-mouse IgG (MedChemExpress Cat# HY-P80952, RRID:AB_3102769; 1:1000). Following counterstaining of nuclei with DAPI-infused mounting media (Biosharp Cat# BL739A), samples were seen using a Zeiss LSM880 Airyscan confocal microscope with calibrated excitation settings: 488 nm laser (BP495-550 nm) for TOM20 and 594 nm laser (LP645 nm) for MitoTracker. VDIMs quantification was performed as follows: Regions of interest were delineated around individual cells, duplicated, and a corresponding mask created for each channel. The 'Erode' command was implemented on the Mitotracker channel, and 'Dilate' on the TOM20 channel to expand its mask by one pixel. The TOM20 mask was subsequently subtracted out from the Mitotracker mask, with the resultant image utilized to assess VDIM count and size via the 'Analyze Particles' plugin 18.

### Ultramicroscopic Pathological Cell Detection

Cultured cells were initially treated with 2.5% glutaraldehyde and subsequently subjected to post-fixation with 1% osmium tetroxide. Following PBS washes, dehydration was conducted using a graded series of acetone and subsequently embedded in Epon-812 epoxy resin using gradient infiltration (acetone/ resin ratios of 3:1 to 1:3). Ultrathin slices were prepared with a Leica EM UC7 ultramicrotome, collected on copper grids, and subjected to double staining with 2% uranyl acetate (15-30 minutes, in the dark) and lead citrate. Grids were analyzed using a Hitachi HT7800 transmission electron microscope at 80 kV, with image acquisition settings refined for optimal organelle contrast.

### Construction of organoid models for BC

Fresh tumor specimens were conveyed in a chilled transport medium of DMEM/F12 (Gibco Cat# 8123512) supplemented with 10 ng/ml EGF (MedChemExpress Cat# HY-P7109), 5% FBS, and 1% penicillin-streptomycin-amphotericin B (Biosharp Cat# BL142A). Following washing in modified Hanks' buffer (Hanks' (Solarbio Cat# H1040) supplemented with 10 μM Y-27632 (MedChemExpress Cat# HY-10071) and 5% FBS), tissues were minced into 1-2 mm³ fragments and subjected to sequential digestion in an enzymatic solution (Collagenase I (Biosharp Cat# EZ7890D412) at a concentration of 10 mg/ml and hyaluronidase (Solarbio Cat# HB030) at 1.64 mg/ml in PBS) at 37 °C for 15 minutes, subsequently treated with TrypLE Express (Gibco Cat# 12605010) at room temperature for 3 minutes. Filtered cell clusters were encapsulated in Matrigel (70% v/v; Mogengel Cat# 0827555) augmented with growth media (DMEM/F12 supplemented with 10 ng/ml EGF, 10 μM Y-27632, 5% FBS, and 1% antibiotics) and deposited as 30 μL domes on 24-well plates. Following Matrigel polymerization at 37 °C for 30 minutes with inverted incubation, 1.5 ml of growth media was introduced and replenished every 72 hours. Organoid production efficiency (50-80%) was assessed using phase-contrast microscopy.

### Construction of PDX model for BC

Fresh tumor specimens were conveyed in ice-cold RPMI-1640 transport media (enhanced with 1% penicillin-streptomycin-amphotericin B and 5% FBS) and mechanically sectioned into 1-3 mm³ pieces. Tissue blocks were subcutaneously transplanted into NOD/SCID/IL2Rγnull (NCG) mice utilizing a Matrigel embedding approach (200 μL of ice-cold Matrigel per graft) via pre-lubricated trocar needles. In the MSCs cohorts, 5×10⁴ human MSCs were first mixed with 200 μL Matrigel, and then co-embedded with tumor pieces before implantation. Tumor passages (P_1_-P_3_) were conducted when grafts attained a volume of 1.0-1.5 cm³, followed by cryopreservation in a solution of 95% FBS and 5% DMSO utilizing programmed freezing techniques. Drug intervention commenced at P_3_ with Farnesoid X receptor (FXR) agonist 3 (MedChemExpress Cat# HY-151932; 10 mg/kg in PBS, q3d) compared to vehicle control. The tumor volume was determined using the formula V=½×L×W², based on measurements obtained using a digital caliper.

### Statistical analysis

The Prism program (GraphPad Software) served as the foundation for the statistics in this study. The independent sample t-test is used for the difference analysis of two samples for quantitative data that follows a normal distribution; the non-parametric test is used for quantitative data that does not follow a normal distribution; the chi-square test is used for counting data; and the log-rank test is used for Kaplan-Meier analysis. Pearson's analysis was used to perform correlation analysis. The threshold for statistical significance was set at *P* < 0.05.

## Results

### The infiltration level of MSCs is closely associated with adverse clinical features of BC, and TIMP1 is the primary cytokine released by MSCs

**Figure 1:**
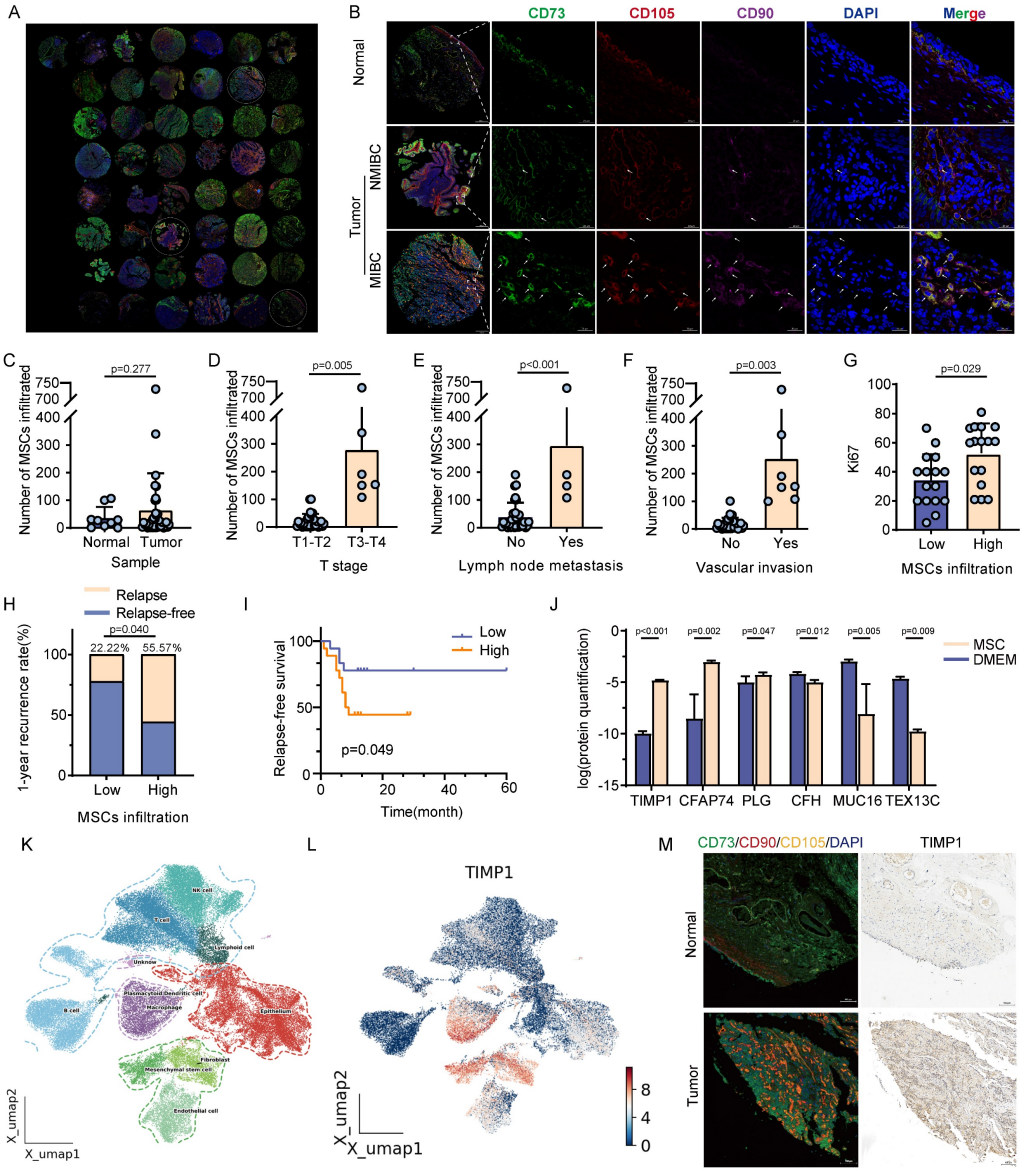
** Analysis of the relationship between MSCs infiltration level and clinical features of BC, and identification of major cytokines secreted by MSCs. (A)** Global view of the BC tissue microarray after mIHC. **(B)** Distribution differences of MSCs in normal tissue, NMIBC, and MIBC samples (9 normal tissues, 36 BCa tissues, 4 non-BCa primary tumor samples). **(C-F)** Comparison of MSCs infiltration levels in BC samples with different clinical features after quantifying tissue samples (n = 36). **(G)** Comparison of Ki-67 positive percentages in high and low MSCs infiltration samples, grouped based on the median MSCs infiltration value (n = 36).** (H)** Tumor recurrence rates within 1 year in patients with high and low MSCs infiltration specimens (n = 36). **(I)** Kaplan-Meier curves for recurrence-free survival in patients with high and low MSCs infiltration samples (n = 36). **(J)** Results of differential protein detection in the supernatant of MSCs culture versus normal uncultured MSCs medium. **(K-L)** Identification of cell types in single-cell genomics of BC tissue samples from public databases, and analysis of TIMP1 expression distribution on the UMAP plot. **(M)** Comparison of mIHC for MSCs-specific markers and IHC for TIMP1 within the same tissue region of normal or tumor tissue samples.

This study performed mIHC analysis on 45 tissue samples obtained over the last year to investigate the association between MSCs and the advancement of BC, utilizing specific MSCs markers (CD73, CD90, and CD105). The infiltration level of MSCs in BC samples was substantially greater than in normal urothelial samples. Furthermore, the infiltration of MSCs was more significant in muscle-invasive bladder cancer (MIBC) compared with non-muscle-invasive bladder cancer (NMIBC) (**Fig. 1A-B**). The quantitative results indicated that the infiltration level of MSCs was markedly increased in samples with advanced clinical stages (T_3-4_), lymph node metastases, and vascular invasion (**Fig. 1C-F**). All bladder urothelial carcinoma samples were classified into high and low MSCs infiltration groups according to the level of infiltration. In BC samples with higher MSCs infiltration, the percentage of Ki-67-positive cells was markedly increased, and patients with high MSCs infiltration experienced a significantly greater tumor recurrence rate within one year post-surgery compared with those with low MSCs infiltration (55.57% vs. 22.22%, *P* < 0.05) (**Fig. 1G-H**). Kaplan-Meier analysis indicated that patients with elevated MSCs infiltration experienced significantly reduced disease-free survival compared with those with low MSCs infiltration (*P* < 0.05) (**Fig. 1I**). Mass spectrometry analysis was carried out on MSCs culture supernatants to seek out cytokines potentially involved in MSCs-mediated tumor growth. The findings indicated TIMP1, CFAP74, PLG, CFH, MUC16, and TEX13C as the most significantly altered cytokines (**Fig. 1J**). Analysis of the GSE 222315 dataset and BC single-cell omics data revealed that TIMP1 was predominantly expressed in MSCs and other non-tumor cells, as indicated by particular markers in single-cell histology (**Fig. 1K-L**). Histological analysis revealed a considerable increase in TIMP1 expression in tissue regions with substantial MSCs infiltration (**Fig. 1M**).

### MSCs promote BC proliferation via activation of the RAP1 pathway

**Figure 2:**
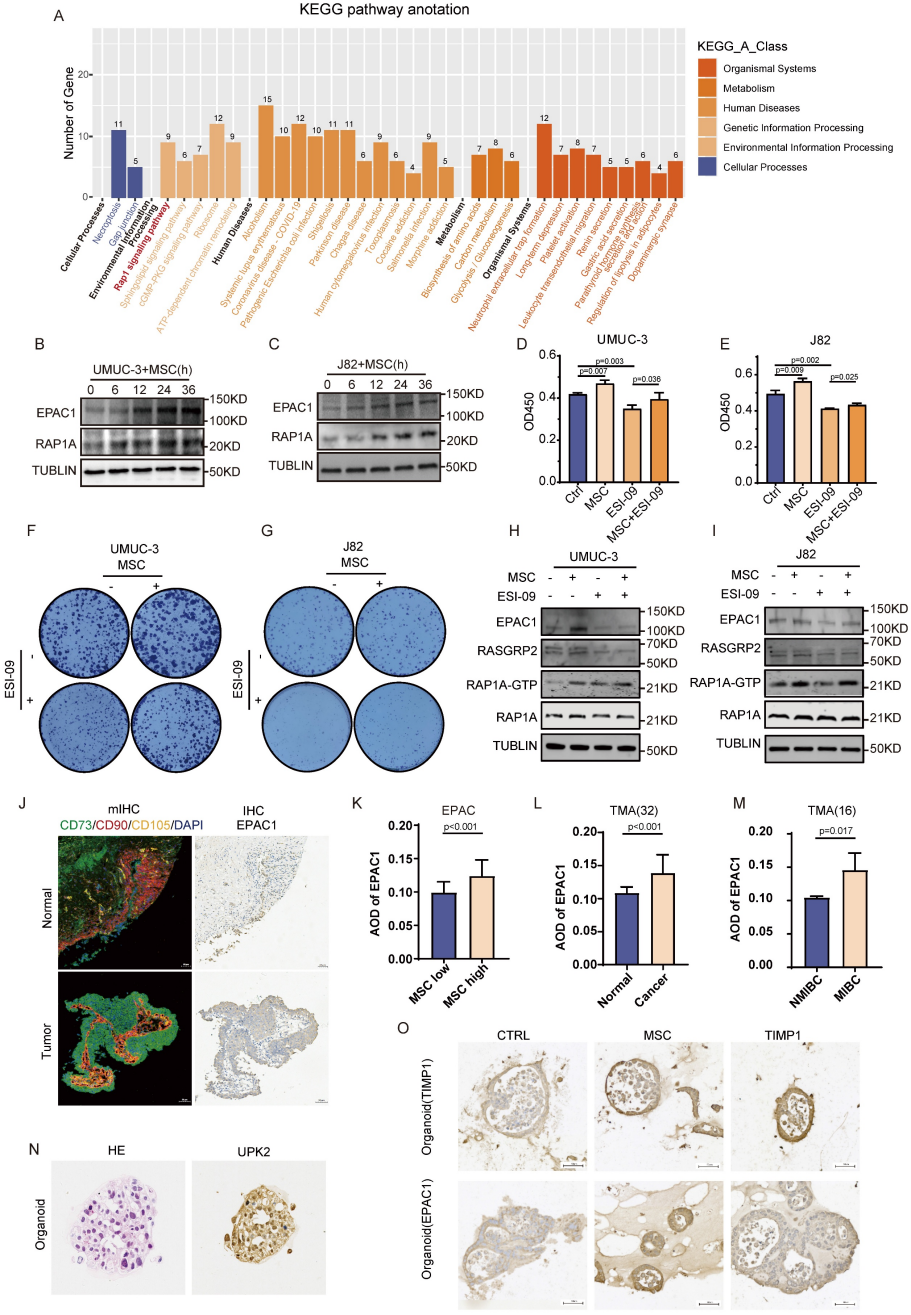
** MSCs promote the proliferation of BC by activating the RAP1 pathway. (A)** KEGG enrichment analysis of differential proteins in UMUC-3 cells detected by mass spectrometry after co-culture with MSCs for 24 hours.** (B-C)** The effect of time-gradient co-culture of UMUC-3 and J82 cells with MSCs on EPAC1 and RAP1 proteins. **(D-E)** CCK-8 experiment results of UMUC-3 and J82 cells after 48 hours of intervention with MSCs-CM and ESI-09 10μM. **(F-G)** The impact of MSCs-CM and ESI-09 10μM on the clone formation of UMUC-3 and J82 cells. **(H-I)** Detection of changes in RAP1 pathway-related proteins in UMUC-3 and J82 cells after 24 hours of intervention with MSCs-CM, ESI-09 10μM, and their combination; RAP1A-GTP protein was obtained through GST-pulldown experiment for subsequent protein detection. **(J)** Comparison of mIHC labelling of MSCs and IHC staining of TIMP1 within identical regions of BC and normal tissue. **(K-M)** Quantitative analysis results of IHC staining of EPAC1 in BC tissue samples with different clinical features. **(N)** HE and IHC staining of the BC marker UPK2 in the constructed organoids. **(O)** IHC staining results of TIMP1 and EPAC1 in samples after intervention with TIMP1 12.5ng/ml and MSCs-CM in the BC organoid model.

The findings above indicate a strong correlation between MSCs and BC advancement. Nonetheless, the specific mechanisms by which MSCs influence alterations in BC remain unclear. Consequently, mass spectrometry is utilized to identify the proteome alterations in BC cells following MSCs intervention. KEGG enrichment analysis found that these proteins were mostly abundant in the RAP1 signaling pathway (**Fig. 2A**). BC cells (UMUC-3 and J82) were co-cultured with MSCs for varying time intervals, revealing that MSCs enhanced the expression of EPAC1 and RAP1A, with a progressive rise in expression over time (**Fig. 2B-C**). The use of the RAP1 pathway inhibitor ESI-09 on BC cells demonstrated that MSCs-CM markedly enhanced the proliferation of UMUC-3 and J82 tumor cells, but ESI-09 impeded tumor growth. The combination of MSCs-CM and ESI-09 resulted in a considerably greater proliferation of tumor cells compared with ESI-09 alone, however, lower than that observed with MSCs-CM alone (*P*<0.05) (**Fig. 2D-G**). The RAP1-GTP protein was acquired under various intervention settings utilizing the RAP1 activity protein GST-pulldown kit. Western blot examination demonstrated that the application of MSCs-CM to BC cells (UMUC-3 and J82) resulted in a considerable upregulation of critical proteins in the RAP1 pathway, including EPAC1, RASGRP2, and the active form of RAP1A (RAP1A-GTP). The intervention of ESI-09 to tumor cells resulted in a marked reduction in the expression of these proteins, but the concurrent intervention of MSCs-CM and ESI-09 led to a considerably elevated expression of these proteins in comparison to ESI-09 alone (**Fig. 2H-I**). In tumor specimens, elevated levels of MSCs infiltration were seen, correlating with increased expression levels of EPAC1 (**Fig. 2J**). The expression of EPAC1 in tumor samples, samples with high MSCs infiltration, and MIBC samples were considerably elevated compared with paracancerous samples, samples with low MSCs infiltration, and NMIBC samples (**Fig. 2K-M**). The engineered BC organoids exhibited a substantial increase in the expression of EPAC1 and TIMP1 when exposed to MSCs-CM (**Fig. 2O**). This indicates that MSCs affect the biological behavior of tumors by activating the RAP1 pathway in BC.

### TIMP1 secreted by MSCs enhances BC proliferation

**Figure 3:**
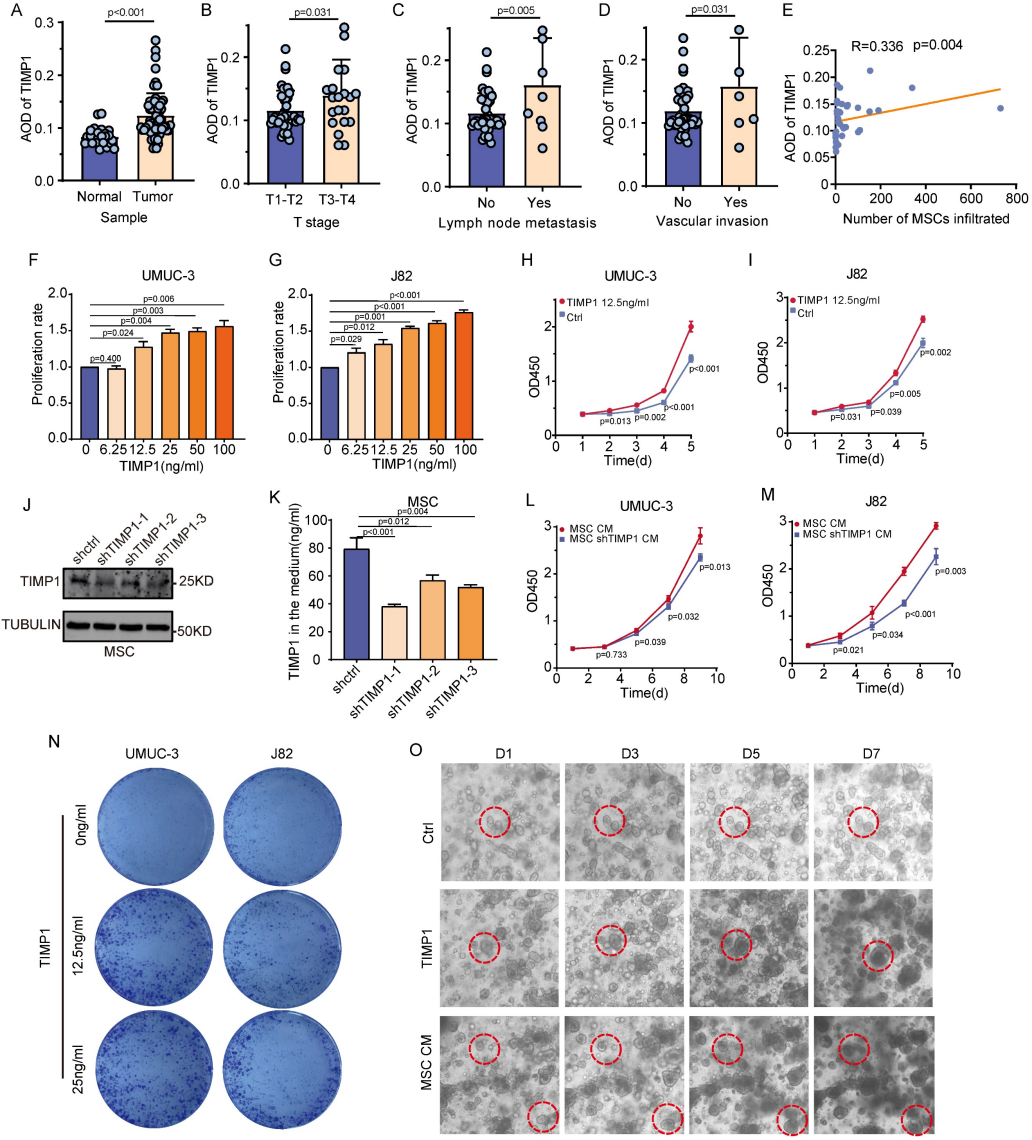
** TIMP1 secreted by MSCs promotes the proliferation of BC. (A-D)** IHC analysis of TIMP1 expression distribution across different clinical features of BC, with quantification of staining results using average optical density values (n = 56). **(E)** Correlation analysis between TIMP1 expression and MSCs infiltration levels in the same samples (n = 36). **(F-G)** Proliferation fold changes of UMUC-3 and J82 cells after 48 hours of intervention with varying concentrations of TIMP1, measured by absorbance values and compared to the control group. **(H-I)** The effect of TIMP1 at 12.5ng/ml on the proliferation fold change of UMUC-3 and J82 cells over a gradient of time. **(J)** TIMP1 expression levels in the constructed MSCs-shTIMP1 cell. **(K)** TIMP1 secretion levels in the culture supernatant of the constructed MSCs-shTIMP1 cell. **(L-M)** The impact of CM from MSCs-shTIMP1 on the proliferation of UMUC-3 and J82 cells. **(N)** Plate clone formation assay after intervention with different concentrations of TIMP1 on UMUC-3 and J82 cells. **(O)** In the constructed BC organoid model, observation of changes in the size of the same organoid within the same field of view after intervention with TIMP1 at 12.5ng/ml and MSCs-CM.

TIMP1 is the primary cytokine released by MSCs; however, it remains uncertain whether it is the principal factor affecting the biological behavior of BC mediated by MSCs. The quantitative analysis of TIMP1 IHC results indicated that TIMP1 expression was markedly increased in samples with advanced clinical stages (T_3-4_), lymph node metastasis, and vascular invasion, revealing a significant positive correlation between TIMP1 expression levels and MSCs infiltration levels (**Fig. 3A-E**). UMUC-3 and J82 cells underwent intervention with recombinant TIMP1 protein for a duration of 48 hours. It was observed that an increase in cytokine concentration corresponded with a considerable rise in the proliferation rate of UMUC-3 and J82 cells (**Fig. 3F-G**). Intervention with 12.5ng/ml of TIMP1 elicited a more significant impact on cell proliferation as the duration of culture increased (*P* < 0.05) (**Fig. 3H-I**). The plate clone creation experiment demonstrated that a TIMP1 concentration over 12.5ng/ml greatly enhanced cell clone generation (**Fig. 3N**). A TIMP1 knockdown lentivirus was subsequently engineered to reduce TIMP1 expression in MSCs, aiming to examine the impact of TIMP1 knockdown on the proliferative effect of MSCs on tumor cells. The protein expression level of TIMP1 in stable cell lines is shown in the figure (**Fig. 3J**). The Elisa data indicated that following the lentiviral knockdown of TIMP1 in MSCs, the TIMP1 concentration in the cell culture supernatant was markedly decreased (*P* < 0.001) (**Fig. 3K**). CM from MSCs with TIMP1 knockdown (MSCs-shTIMP1) was harvested and utilized to treat UMUC-3 and J82 cells. The findings indicated that, relative to the standard MSCs-CM, the MSCs-CM with TIMP1 knockdown decreased cell proliferation, with this impact becoming increasingly pronounced over time (*P* < 0.05) (**Fig. 3L-M**). BC organoids were treated with regular media containing TIMP1 and grown in organoid medium supplemented with MSCs. The proliferation rate of organoids, influenced by TIMP1 and MSCs-CM, dramatically increased, resulting in larger organoid sizes (**Fig. 3O**). This demonstrates that TIMP1 secreted by MSCs can increase the growth of BC.

### TIMP1 was identified as closely associated with the invasion of MSCs in publicly available data and clinical urine samples

**Figure 4:**
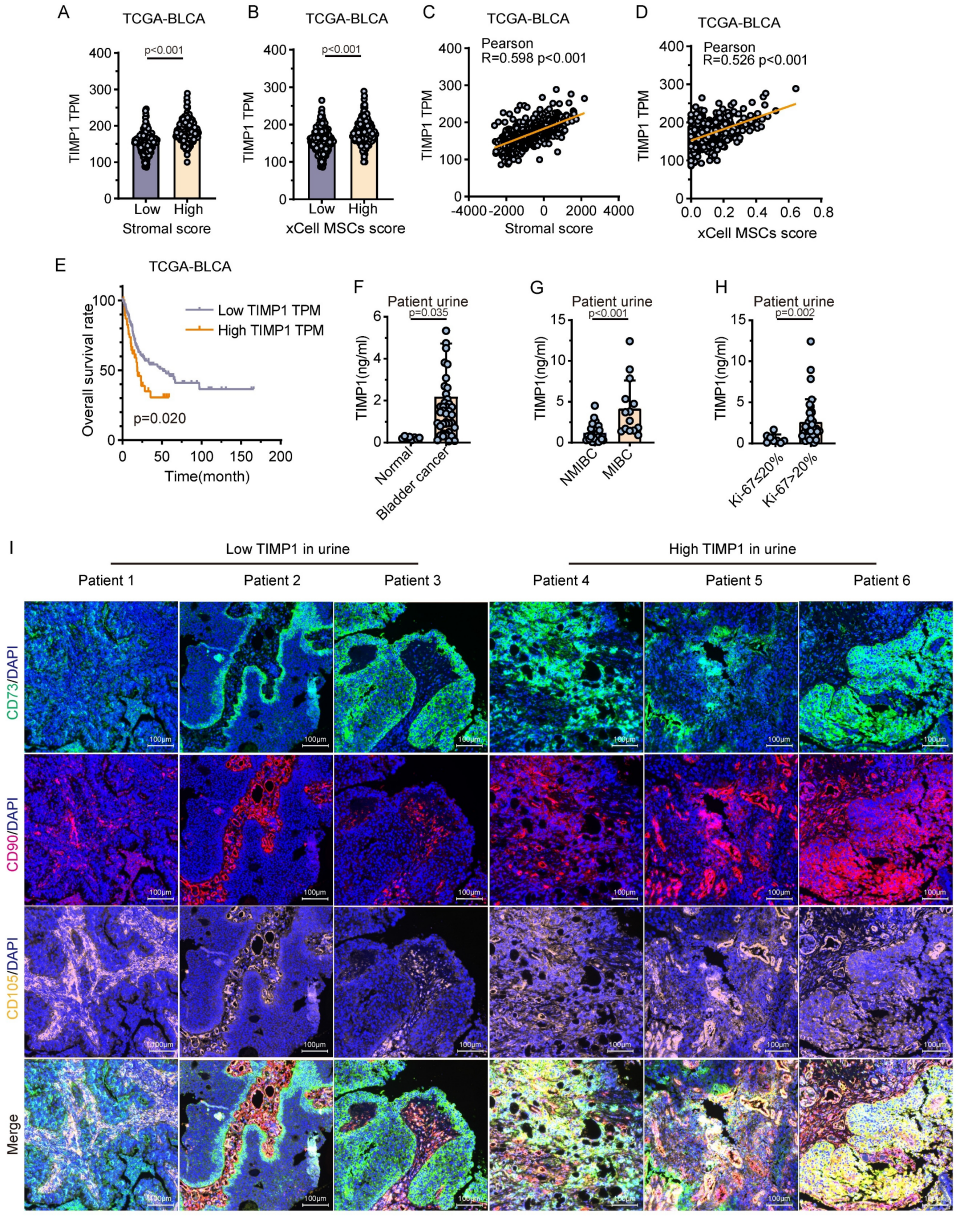
** Validation of TIMP1 in public data and clinical urine samples. (A)** Expression levels of TIMP1 in high and low stromal infiltration samples based on the median stromal score. **(B)** Expression levels of TIMP1 in high and low MSCs infiltration samples based on the median MSCs score. **(C-D)** Correlation analysis between TIMP1 expression levels in tissues and stromal scores, as well as MSCs scores. **(E)** Kaplan-Meier analysis of overall survival time in samples with high and low TIMP1 expression based on the optimal cut-off value. **(F)** Differences in TIMP1 expression levels in urine between tumor patients and non-tumor patients (n = 50). **(G-H)** Differences in TIMP1 expression levels in urine among BC patients with different clinical features (n = 42). **(I)** mIHC of MSCs-specific markers in tissue samples from patients with high and low TIMP1 expression levels in urine.

Analysis of the TCGA public database indicated that TIMP1 gene expression was markedly elevated in tissue samples exhibiting high stroma or high MSCs scores in comparison with those with low stroma or low MSCs scores (*P* < 0.001). Additionally, TIMP1 gene expression exhibited a notable positive connection with both stroma score and MSCs score (**Fig. 4A-D**). The Kaplan-Meier analysis of overall survival showed that the survival time of patients with elevated expression of TIMP1 was significantly shortened (**Fig. 4E**). Urine samples from 50 patients were obtained for TIMP1 detection, revealing that TIMP1 levels were markedly elevated in the urine of BC patients (n = 42) relative to non-tumor patients (n = 8) (*P* < 0.05) (**Fig. 4F**). In tumor patients, urine samples from individuals with MIBC, high grade and Ki-67 positive cell counts over 20% exhibited substantially elevated TIMP1 levels compared with those with NMIBC (*P* < 0.001), low grade (*P* < 0.01) and patients with Ki-67 positive cell counts of 20% or less (*P* < 0.01) (**Fig. 4G-H, Table S1**). mIHC for MSCs was conducted on BC tissues from six patients. The findings indicated that tissue samples from patients exhibiting elevated urine TIMP1 levels demonstrated significantly greater MSCs infiltration compared with those with reduced urine TIMP1 levels (**Fig. 4I**). The strong association between TIMP1 and MSCs has been confirmed by public datasets and clinical urine samples, demonstrating that TIMP1 is one of the main substances that mediate the functions of MSCs.

### TIMP1 secreted by MSCs activates the RAP1 signaling pathway via cMet

**Figure 5:**
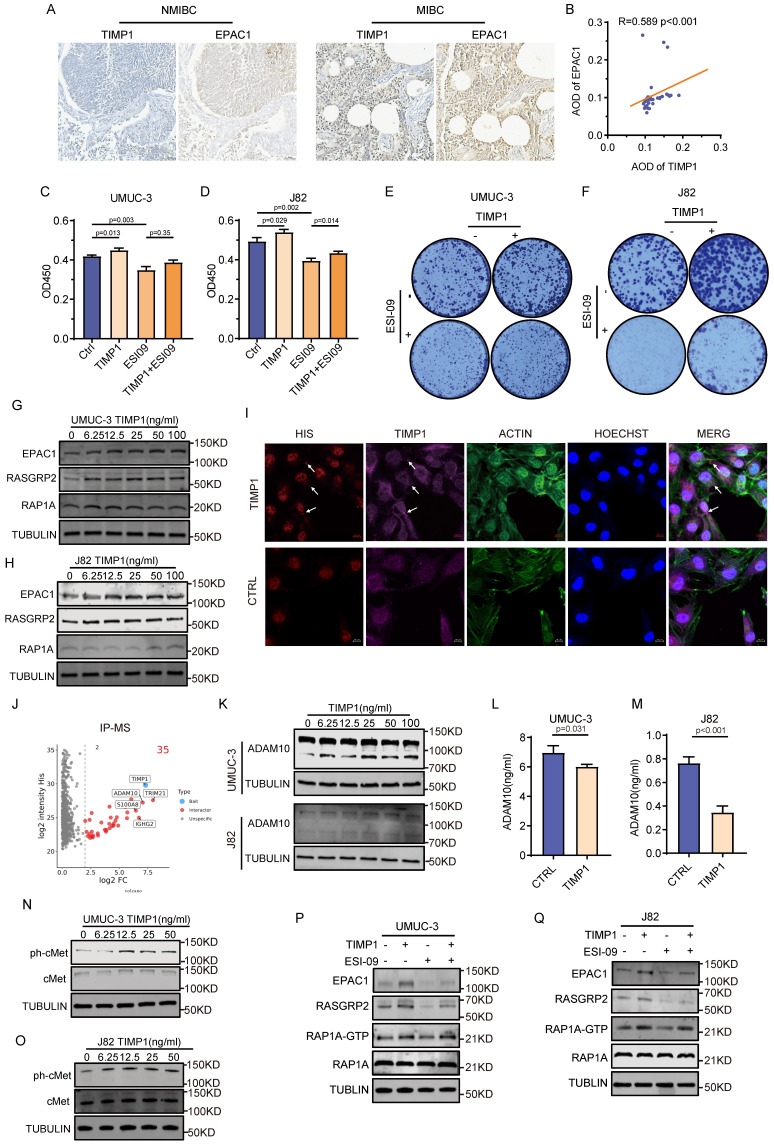
** TIMP1 secreted by MSCs activates the RAP1 signaling pathway via cMet. (A)** Comparison of IHC staining results for TIMP1 and EPAC1 within the same tissue region of NMIBC and MIBC samples. **(B)** Correlation analysis of quantitative results from TIMP1 and EPAC1 IHC staining. **(C-F)** CCK-8 results of UMUC-3 and J82 cells after 48 hours of intervention with TIMP1 12.5ng/ml, ESI-09 10μM, and their combination; as well as the impact of these intervention conditions on the clone formation of UMUC-3 and J82 cells. **(G-H)** Changes in key proteins of the RAP1 pathway in UMUC-3 and J82 cells after 24 hours of intervention with varying concentrations of TIMP1. **(I)** Following 24 hours of stimulation with TIMP1 at a concentration of 12.5ng/ml in UMUC-3 cells, mouse His and rabbit TIMP1 primary antibodies were employed to bind to recombinant TIMP1-his protein, while the cytoskeleton was labeled using ACTIN probes.** (J)** Proteins interacting with TIMP1 identified by IP mass spectrometry after incubation of total protein from UMUC-3 cells with exogenous TIMP1-his protein.** (K)** Changes in intracellular ADAM10 expression levels in UMUC-3 and J82 cells after 24 hours of intervention with varying concentrations of TIMP1. **(L-M)** Changes in ADAM10 levels in the culture supernatant detected by ELISA after 24 hours of intervention with medium containing 12.5ng/ml TIMP1 in UMUC-3 and J82 cells. **(N-O)** Changes in key protein levels of the cMet signaling pathway in UMUC-3 and J82 cells after 24 hours of intervention with medium containing varying concentrations of TIMP1. **(P-Q)** Changes in RAP1 pathway-related proteins in UMUC-3 and J82 cells after 24 hours of intervention with medium containing TIMP1 at 12.5ng/ml, ESI-09 10μM, and their combination.

TIMP1 is strongly associated with the development of BC; hence, this study investigated whether TIMP1 released by MSCs influences alterations in the RAP1 pathway. Histological correlation analysis revealed a substantial positive association between TIMP1 and EPAC1 expression levels (**Fig. 5A-B**). The impact of the simultaneous intervention of TIMP1 and ESI-09 on the growth of BC cells mirrors that of MSCs in conjunction with ESI-09 (**Fig. 5C-F**). Intervention of BC cells with various concentrations of TIMP1 resulted in a vital elevation in the expression levels of critical proteins within the RAP1 pathway, specifically EPAC1, RASGRP2, and RAP1A. The expression level of EPAC1 rose progressively with rising TIMP1 concentration in the medium, demonstrating a concentration-dependent impact (**Fig. 5G-H**). Immunofluorescence research demonstrated that exogenous TIMP1-his protein was mostly localized in the cell membrane and cytoplasm following intervention (**Fig. 5I**). Consequently, complete protein extracts from UMUC-3 cells were treated with exogenous TIMP1-his, followed by HIS tag pull-down and subsequent protein mass spectrometry analysis. The findings indicated that TRIM21, ADAM10, and other proteins interacted with TIMP1 (**Fig. 5J, Fig.S2A-E**). The intervention of UMUC-3 and J82 cells with varying doses of TIMP1 demonstrated that the mature form of ADAM10 in the cytoplasm of UMUC-3 cells escalated with higher TIMP1 concentrations, whereas a less pronounced trend was noted in J82 cells (**Fig. 5K**). Nevertheless, the concentration of ADAM10 in the culture supernatant was markedly reduced, although there was no substantial alteration in the transcriptomic level of ADAM10 (**Fig. 5L-M, Fig. S2F-G**). Intervention of cells with varying concentrations of TIMP1 and subsequent analysis of cMet signaling pathway-related proteins revealed a significant increase in the phosphorylation level of cMet protein following TIMP1 intervention of bladder tumor cells, peaking at a TIMP1 concentration of 12.5 ng/ml, whereas non-phosphorylated cMet exhibited no notable change (**Fig. 5N-O**). The amalgamation of ESI-09 and TIMP1 counteracted the suppressive influence of ESI-09 on the expression of EPAC1, RASGRP2, and RAP1A-GTP (**Fig. 5P-Q**). The above results indicate that TIMP1 enters the intracellular space and binds to ADAM10. By reducing the extracellular secretion of ADAM10, it avoids the cleavage of cMet on the cell membrane, thereby promoting the phosphorylation of cMet and ultimately leading to the activation of the RAP1 pathway.

### TIMP1 secreted by MSCs stimulates the formation of VDIMs via the Rap1 signaling pathway

**Figure 6:**
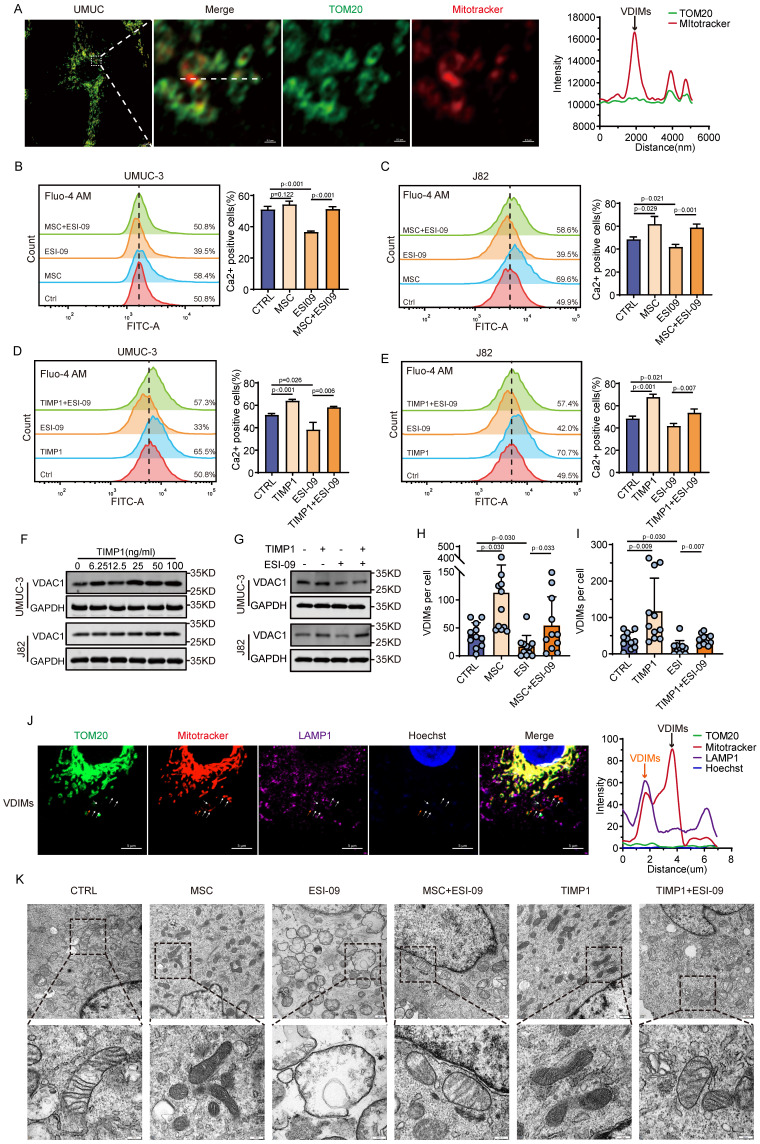
** TIMP1 promotes the formation of VDIMs through the Rap1 signaling pathway. (A)** Schematic diagram of VDIMs in UMUC-3 cells, with colocalization curves confirming MitoTracker (+)/ TOM20 (-). **(B-E)** Flow cytometry detection of intracellular Ca^2+^ levels using the Fluo-4/AM probe in UMUC-3 and J82 cells after 24 hours of treatment with MSCs -CM/TIMP1 combined with ESI-09.** (F-G)** Changes in intracellular VDAC1 expression levels in UMUC-3 and J82 cells after 24 hours of intervention with varying concentrations of TIMP1 and TIMP1 combined with ESI-09. **(H-I)** Effects of MSCs-CM/TIMP1 combined with ESI-09 on the formation of VDIMs in UMUC-3 cells after 24 hours. **(J)** Fluorescence imaging and colocalization curves of VDIMs and the lysosomal marker LAMP1 in UMUC-3 cells, with orange representing VDIMs localized to lysosomes and black representing VDIMs not localized to lysosomes. **(K)** Morphology of mitochondria observed under an electron microscope in UMUC-3 cells after 24 hours of treatment with MSCs-CM/TIMP1 combined with ESI-09.

VDIMs act as a mechanism for mitochondrial quality control and can influence mitochondrial homeostasis inside cell. This study further investigates the influence of MSCs and TIMP1 on the formation of VDIMs. Airyscan confocal imaging of UMUC-3 cells demonstrated the existence of VDIMs (MitoTracker (+)/TOM20 (-)) in BC (**Fig. 6A**). The primary driver for VDIMs formation is alterations in intracellular Ca^2+^ levels. Flow cytometry analysis revealed that the cytoplasmic Ca^2+^ concentration was markedly elevated in J82 cells exposed to MSCs-CM and TIMP1 in comparison with the control group. ESI-09 markedly decreased intracellular Ca^2+^ concentrations at a specific dosage, however the combination of interventions led to elevated cytoplasmic Ca^2+^ levels relative to ESI-09 alone (**Fig. 6B-E**). The quantitative study of VDIMs numbers indicated a correlation with fluctuations in intracellular calcium levels (**Fig. 6H-I, Fig.S3**). VDAC1 is crucial to the creation of VDIMs. The intervention of tumor cells with varying amounts of TIMP1 showed that elevated TIMP1 levels greatly enhanced VDAC1 expression, whereas ESI-09 decreased VDAC1 expression (**Fig. 6F-G**). Subsequent immunofluorescence investigation demonstrated the co-localization of generated VDIMs with the lysosomal marker LAMP1 (**Fig. 6J**). Ultrastructural pathological analysis of cellular mitochondria using electron microscopy revealed several high electron-density granular substances inside the mitochondrial matrix of tumor cells subjected to MSCs and TIMP1 intervention. Tumor cells subjected to ESI-09 exhibited enlarged mitochondria with reduced lamellar cristae and an absence of typical mitochondrial architecture. The amalgamation of MSCs and TIMP1 with ESI-09 markedly enhanced mitochondrial architecture and preserved mitochondrial function (**Fig. 6K**). This suggests that MSCs-secreted TIMP1 maintains mitochondrial quality control, stimulates the production of VDIMs via the RAP1 pathway, and is crucial for sustaining cell development.

### TIMP1 inhibitors inhibit the activation of the RAP1 signaling pathway in BC via MSCs

**Figure 7:**
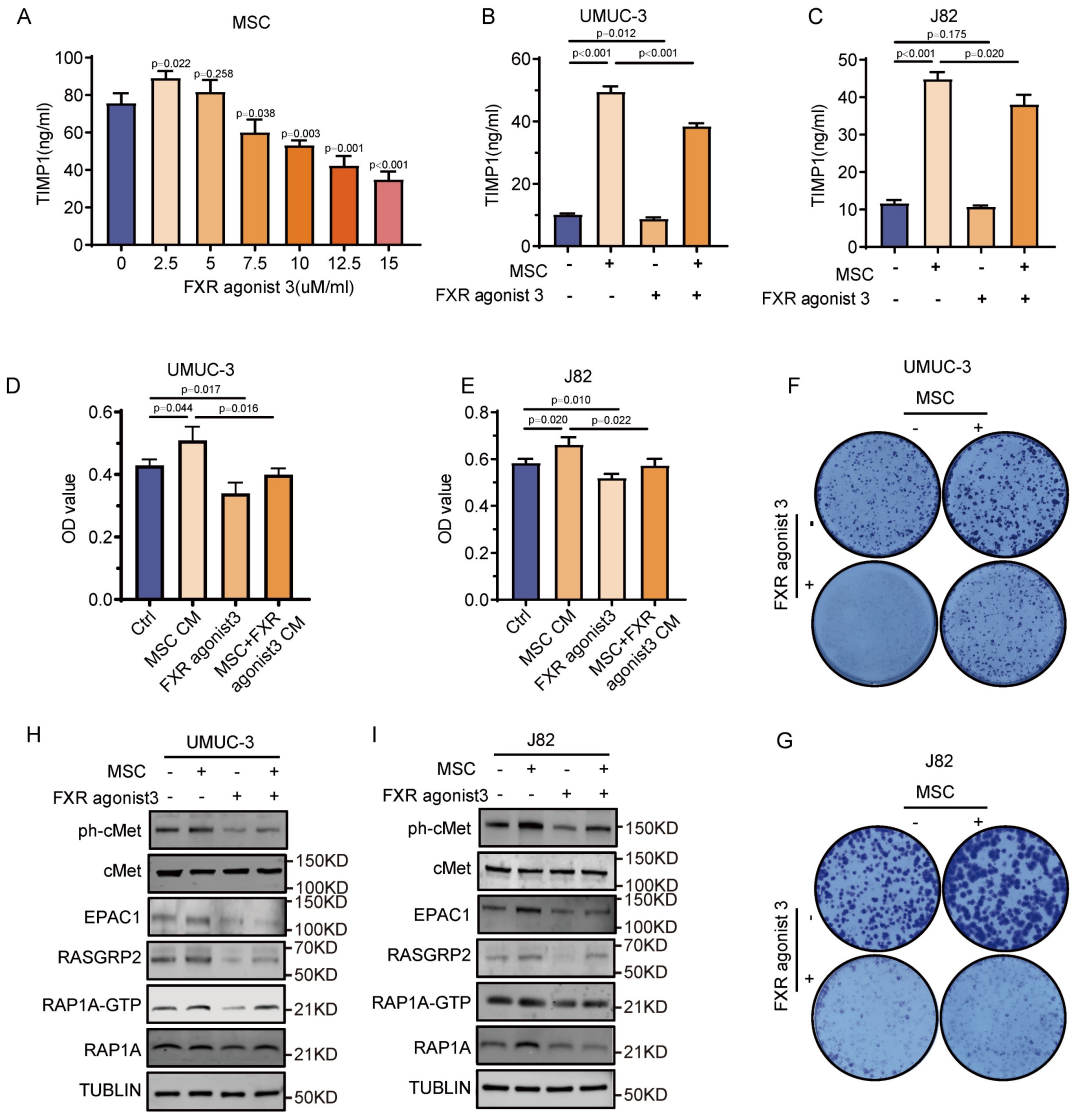
** Inhibition of the RAP1 signaling pathway in BC via intervention in MSCs with FXR agonist 3. (A)** ELISA detection of TIMP1 levels in the culture supernatant after 24 hours of intervention with varying concentrations of FXR agonist 3 in MSCs. **(B-C)** ELISA detection of TIMP1 secretion levels in the culture supernatant after 24 hours of co-culture of MSCs with UMUC-3 and J82 cells in the presence of FXR agonist 3. **(D-G)** Effects of MSCs-CM, FXR agonist 3, and CM from MSCs pretreated with FXR agonist 3 on the proliferation and clonal formation of UMUC-3 and J82 cells after 48 hours of treatment. **(H-I)** Impact of MSCs-CM, FXR agonist 3, and CM from MSCs pretreated with FXR agonist 3 on key proteins of the intratumoral cMet-RAP1 pathway in UMUC-3 and J82 cells after 24 hours of treatment.

An in-depth study is carried out to assess if TIMP1 inhibitors may reverse the tumor-promoting impact of MSCs, with the goal of identifying the mechanism by which TIMP1 released by MSCs promotes BC. The intervention of MSCs with different doses of the TIMP1 inhibitor, FXR agonist 3, showed that at a concentration of 2.5 μM, FXR agonist 3 enhanced TIMP1 secretion. At doses of 7.5 μM or above, it effectively suppressed TIMP1 secretion, resulting in a considerable reduction of TIMP1 levels in the culture supernatant as the concentrations of FXR agonist 3 increased (**Fig. 7A**). In a co-culture system of mesenchymal stem cells and BC cells, a concentration of 10μM FXR agonist 3 markedly decreased the amount of TIMP1 in the culture supernatant (**Fig. 7B-C**). Cell proliferation assays indicated that CM from MSCs treated with FXR agonist 3 diminished the proliferative impact of MSCs-CM, while FXR agonist 3 alone also markedly reduced tumor cell proliferation (**Fig. 7D-G**). Western blot study data demonstrated that MSCs-CM enhanced the production of phosphorylated cMet protein and critical proteins in the RAP1 pathway, including EPAC1, RASGRP2, and RAP1-GTP. The intervention of UMUC-3 and J82 cells with 10μM FXR agonist 3 resulted in a comparable reduction in the expression of critical proteins in the cMet-RAP1 pathway inside the tumor cells. Nonetheless, when exposed to conditioned media from MSCs treated with FXR agonist 3, the expression of these proteins was markedly reduced relative to the normal MSCs-CM group, although remained elevated compared to the FXR agonist 3-only group (**Fig. 7H-I**). *In vitro*, FXR agonist 3 reduced the activation of the cMet-RAP1 signaling axis and prevented BC from proliferating due to MSCs.

### TIMP1 inhibitors can impede tumor growth through MSCs *in vivo*

**Figure 8:**
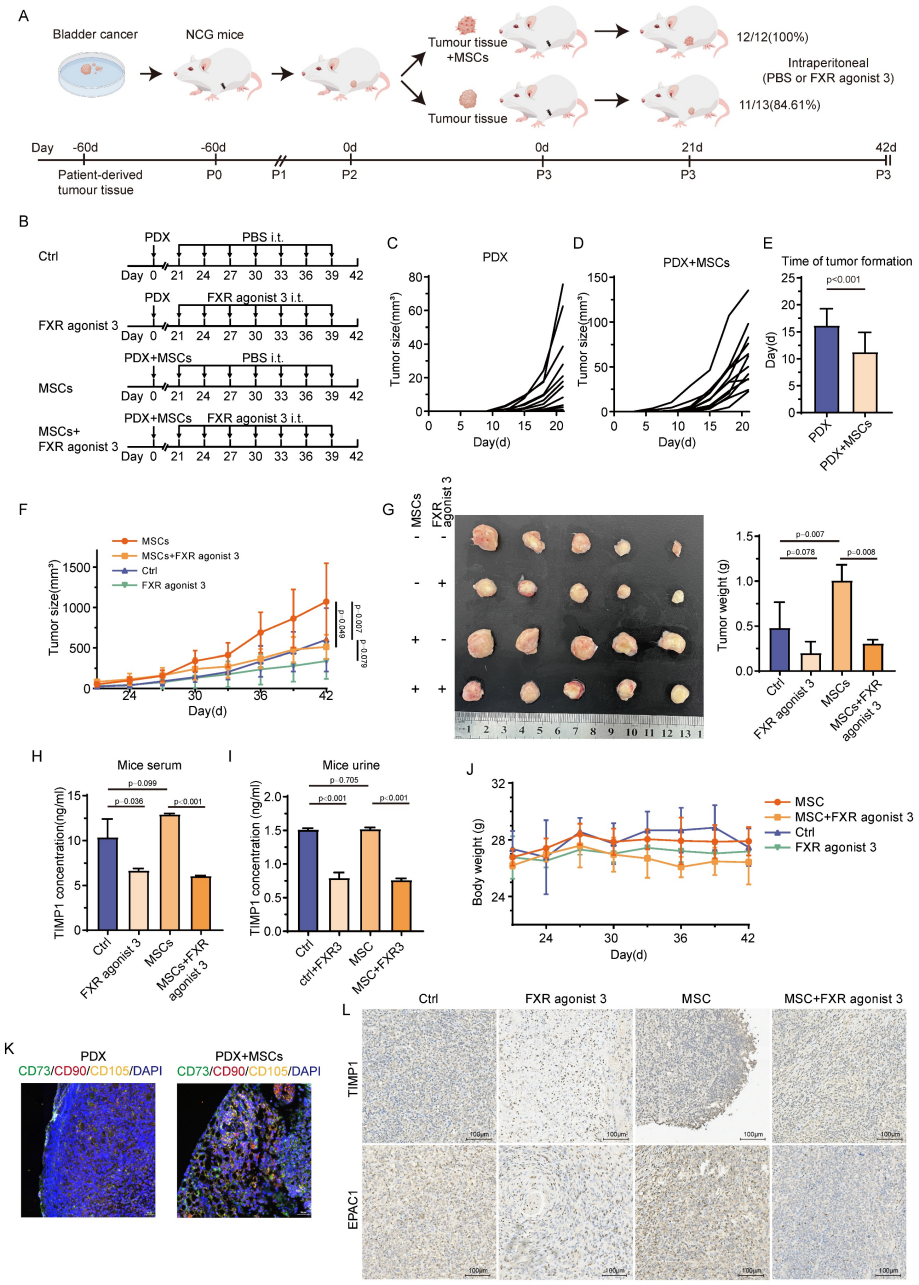
** TIMP1 inhibitor suppresses tumor proliferation by MSCs *in vivo*. (A)** Schematic diagram of the PDX model construction. **(B)** Drug intervention schemes and the timeline for different groups. **(C-D)** Tumor growth curves before drug intervention in mice without MSCs and with MSCs. **E,** Differences in tumor formation time between mice without MSCs and with MSCs (criterion: palpable, hard, clearly defined nodule with a diameter of approximately 2mm). **F,** Tumor growth curves during drug intervention in mice from the CTRL group, FXR agonist 3 group, MSCs group, and MSCs combined with FXR agonist 3 group. **G,** Tumor size and weight distribution at the experimental endpoint for mice in different groups. **H-I,** ELISA detection of TIMP1 secretion levels in serum and urine of mice from different groups at the experimental endpoint. **J,** Weight change trends during drug intervention in mice from different groups. **K,** mIHC staining of tumor tissues based on MSCs markers. **L,** Changes in TIMP1 and EPAC1 protein levels in tumor samples from mice in different groups.

This study constructed a PDX mice model from BC patients and assessed the success rate of tumors 21 days after implantation to assess the association between MSCs and the development of BC in actual clinical practice. The establishment success rate of the PDX model was greater in mice with tumors combined with MSCs than in those without MSCs (100% vs. 83.61%) (**Fig. 8A**). The image illustrates the PDX construction modalities and drug intervention schedules for several groups (**Fig. 8B**). Tumors were palpable earlier, and the duration of tumor growth was considerably reduced in mice receiving MSCs compared with those without (**Fig. 8C-E**). Each cohort of mice was uniformly partitioned into two subgroups according to tumor size, culminating in four groups (20 mice in total), which underwent distinct interventions. The study revealed that in both groups of mice undergoing MSCs intervention, the tumor formation rate was markedly reduced in those administered FXR agonist 3 relative to those treated with PBS (*P* < 0.05). In the two groups of mice without MSCs intervention, the tumor volume in the drug-treated group was less than that in the PBS-treated group; however, the difference was not statistically significant (*P*>0.05) (**Fig. 8F**). **Figure 8G** displays the tumor diameters in each group, with comparable findings in tumor weight and growth trajectory. The examination of TIMP1 levels in the serum and urine of the four mouse groups indicated that the FXR agonist 3 intervention decreased TIMP1 expression in both serum and urine, irrespective of MSCs delivery (**Fig. 8H-I**). In tumor tissue samples from PDX mice subjected to MSCs intervention, MSCs infiltration was noted in the peripheral regions of the tumor tissue (**Fig. 8K**). MSCs enhanced the expression of TIMP1 and EPAC1 in tumor tissue, whereas the FXR agonist 3 reduced their expression levels (**Fig. 8L**). Throughout the pharmaceutical intervention phase, there were no notable alterations in organ damage or body weight among any of the four groups of mice (**Fig. 8J, Fig.S4**). No significant differences in hematopoietic function and serum biochemical parameters were observed in mice treated with FXR agonist 3 (**Table S2**). This indicates that TIMP1 inhibitors can inhibit the promoting effect of MSCS on BC and have good biological safety.

## Discussion

Stromal cells inside the bladder TME are pivotal in tumor growth, metastasis, drug resistance, and personalized treatment by remodeling the microenvironment and engaging with tumor cells 27, 28. In previous studies, we discovered that the small molecule metabolites secreted by MSCs could promote mitochondrial autophagy via the AMPK pathway in order to preserve mitochondrial homeostasis 4. The present research assessed the infiltration level of MSCs in BC tissues and revealed that patients with higher MSCs infiltration exhibited inferior clinical features and reduced recurrence-free survival time. Additionally, the primary cytokine TIMP1 released by MSCs influences the biological activity of BC via the cMet-RAP1 axis. More significantly, our research revealed a novel mitochondrial quality control mechanism in cancers and demonstrated that activation of the RAP1 signaling pathway might preserve mitochondrial homeostasis through promoting the formation of VDIMs in BC mitochondria.

Clinical samples demonstrated an important positive relationship between the infiltration level of MSCs and the expression of TIMP1 in tissues and urine, which was closely associated with adverse clinical features and prognosis. As a multifunctional cytokine, TIMP1 exhibits markedly elevated expression in the majority of cancer tissues and peripheral blood, with the exception of male-specific malignancies (e.g., prostate cancer, testicular cancer, and epididymal carcinoma) 7. Current studies have confirmed that it can serve as a biomarker for the detection of various tumors including lung cancer and pancreatic cancer, as well as for predicting treatment response in ovarian cancer and glioma 6, 8, 29-31. Previous validation studies have reported that urinary TIMP1 is closely associated with the clinical characteristics of bladder cancer, and our research further demonstrates that urinary TIMP1 is also correlated with the infiltration level of MSCs 32. However, further investigations are still required to establish its clinical validity as a biomarker for BC detection.

TIMP1's structural research in recent years has revealed that all of the MMPs' inhibitory sites are located in the N-terminal domain, whereas the C-terminal region binds to signal receptors that promote cell proliferation, including CD63, LRP1, and CD82 33-35. TIMP1 and CD63 expression followed the same trend in tumor cells. TIMP1 interacts with the extensive extracellular loop of CD63 via the C-terminal domain, thereby relaying extracellular signals to intracellular pathways and promoting tumor growth 36-38. In diverse cellular contexts, including breast cancer, TIMP1 enhances the expression of its receptor CD63 through synergistic interactions with CD63 and integrin β1, subsequently activating STAT3 and ERK1/2, thereby facilitating cellular proliferation and migration 36, 39, 40. Previous studies of TIMP1 in tumors focused on the tumor cells alone, neglecting the impact of microenvironmental cells. Our research shows that TIMP1 is predominantly secreted by stromal cells, including MSCs and fibroblasts, inside the TME, rather than by the tumor cells themselves. In addition, MSCs could stimulate the RAP1 pathway in BC cells, and the expression of TIMP1 exhibited a substantial association with the expression of EPAC1, a crucial protein in the RAP1 pathway. The activation of the RAP1 pathway in various cells within the TME has multiple effects on the tumor, either preventing or encouraging cancer progression. In non-tumor cells, such as T cells in the microenvironment, external signals can activate the RAP1 pathway, which subsequently activates integrins, enhances the affinity of leukocyte function-associated antigen-1, and facilitates the infiltration of immune cells into tumor cells to exert a cytotoxic effect 41. Conversely, activated RAP1 protein is abundantly produced in tumor cells, functioning as a crucial mediator for relaying diverse extracellular signals into the cell. The activated RAP1 protein can stimulate tumor-associated signaling pathways, including ERK, MAPK, and Src/FAK 42. Our research demonstrates that TIMP1 can activate the RAP1 pathway in bladder tumor cells, hence promoting tumor growth, revealing a novel mechanism of effect.

This study was conducted using IP mass spectrometry to examine the influence of TIMP1 on the RAP1 pathway, identifying ADAM10 as a key protein interacting with TIMP1. ADAM10 is a member of the a disintegrin and metalloproteinase family and has a catalytic domain that typically cleaves transmembrane protein substrates adjacent to the cell membrane. It regulates cell-cell and cell-matrix interactions, as well as cell growth, differentiation, migration, and receptor-ligand signaling 43, 44. Research indicates that TIMP1 interacts with ADAM10, inhibiting its MMPs activity, which leads to decreased ectodomain shedding of cMet and elevated phosphorylation levels of cMet 45, 46. The activation of cMet can swiftly result in an elevation of the second messenger cAMP and downstream EPAC1, subsequently activating the RAP1 protein, thus facilitating its signal transduction activity 47, 48. Consequently, ADAM10 may serve as an intermediary in the mechanism by which TIMP1 impacts RAP1 through modulation of the cMet pathway. This study revealed that the application of gradient concentrations of TIMP1 to tumor cells resulted in a considerable rise in the mature form of ADAM10 within the cells, although TIMP1 did not influence the transcriptional level of ADAM10. Analysis of alterations in ADAM10 within the extracellular supernatant illustrated a substantial reduction of ADAM10 following TIMP1 intervention. This indicates that TIMP1's interaction to ADAM10 within the cells diminishes its extracellular release and elevates the intracellular levels of ADAM10. The study revealed that the shedding enzyme activity of ADAM10 has a dual function in tumor cell formation, contingent upon its shedding targets. On one side, it may cleave Notch to activate the Notch signaling pathway and cleave EGF to stimulate EGFR receptors, so exerting a tumor-promoting impact 49, 50. Conversely, when it cleaves cMet, it may perform the contrary role as previously stated.

Previous studies have demonstrated that MSCs can maintain mitochondrial homeostasis by increasing mitochondrial autophagy 4. Based on earlier studies, this study was further shown that MSCs can selectively clear the impaired mitochondrial inner membrane, produce VDIMs and be phagocytosis by lysosomes, ultimately maintaining mitochondrial homeostasis. Damage to the mitochondrial cristae results in the production of reactive oxygen species, which can activate the channel on lysosomes adjacent to the mitochondria, leading to the release of Ca^2+^ into the cytoplasm 51. This results in the oligomerization of voltage-gated anion channels on the outer mitochondrial membrane, permitting the escape of damaged inner mitochondrial membrane fragments through these pores, thereby forming VDIMs, which are subsequently engulfed by neighboring lysosomes 18. This research represents the first demonstration that the RAP1 pathway promotes the formation of VDIMs and regulates mitochondrial quality control. The RAP1 pathway and cytoplasmic Ca^2+^ levels are interrelated; Ca^2+^ functions as a second messenger that modulates the activation of the RAP1 pathway, while the activation of the RAP1 pathway can also impact Ca^2+^ concentrations 52. Agonists of G protein-coupled receptors and tyrosine kinase receptors on the cell membrane employ inositol 1,4,5-trisphosphate to facilitate the release of Ca^2+^ from the endoplasmic reticulum, thereby enhancing their signal transduction and activating the downstream RAP1 pathway 53. Elevated Ca^2+^ concentrations induce VDAC1 to oligomerize and form large pores, thereby releasing various components, including mtDNA and vesicles, which promote substance exchange between the mitochondria and the external environment, and establish conditions conducive to the formation of VDIMs 54. The produced VDIMs colocalize with the lysosomal marker LAMP1, and morphological alterations in the mitochondria were verified by electron microscopy. This suggests that BC can modulate oxidative phosphorylation during cellular proliferation via this innovative mode of mitochondrial quality control.

Present study discovered that the release of TIMP1 by MSCs could promote the advancement of BC. Subsequently, utilizing the TIMP1 inhibitor (FXR agonist 3) to intervene in the co-culture system of MSCs and TIMP1 revealed a significant reversal of the proliferation-enhancing effect of MSCs on BC. FXR agonist 3 functions as an agonist of the FXR, which is chiefly implicated in the metabolism of glucose, lipids, bile acids, and cholesterol. It is intricately connected to the initiation and advancement of conditions such as cholestasis, atherosclerosis, inflammatory bowel disease, non-alcoholic fatty liver disease, and malignant neoplasms 55, 56. In liver cancer tissues, FXR expression is markedly diminished relative to surrounding normal tissues; transfection of the FXR gene into human liver cancer cells substantially suppresses tumor cell growth both *in vitro* and *in vivo* 57. FXR agonist 3 may bind to the FXR receptor, inhibiting the transcriptional production of COL1A1, TGF-β1, α-SMA, and TIMP1, hence influencing protein levels and demonstrating anti-fibrotic effect 58. *In vitro* studies indicate that FXR agonist 3 can markedly reverse the proliferative effects of MSCs on bladder tumor cells and influence the modifications of the cMet-RAP1 pathway. In the established bladder cancer PDX model, we noted that FXR agonist 3 could reduce the tumor-promoting effects of MSCs; however, in the absence of MSCs intervention, FXR agonist 3 did not significantly diminish tumor size. This suggests that TIMP1 released by MSCs promoted significantly tumor development. In experiments on animals, FXR agonist 3 exhibited favorable safety, thereby establishing a basis for the development of potent anti-tumor therapeutics aimed at the impacts of MSCs.

This study reveals that TIMP1 is a predominant cytokine released by MSCs, and the infiltration of MSCs in tissues, along with TIMP1 expression levels in tissues and urine, correlates significantly with the clinical features of patients. At the cellular level, TIMP1 released by MSCs stimulates the RAP1 signaling pathway via the cMet pathway, influencing tumor cell proliferation and mitochondrial quality control. The TIMP1 inhibitor FXR agonist 3 may counteract the proliferative influence of MSCs on BC cells, positioning it as a prospective intervention alternative for BC. This study has elucidated a novel mechanism by which MSCs in the TME facilitate the advancement of BC, establishing a basis for investigating personalized MSCs-targeted therapies for BC patients.

## Supplementary Material

Supplementary methods, figures and tables.

## Data Availability

All data generated or analyzed during this study are included in this published article.
