# Supplementary material for: TIMP1 Derived from Mesenchymal Stem Cells Promotes Bladder Cancer Progression by Regulating the Formation of VDIMs through the RAP1 Pathway
Source: Int J Biol Sci. 2026 Mar 17;22(7):3322–41. doi: 10.7150/ijbs.130720 (PMC13085890; doi:10.7150/ijbs.130720)
Supplement: Supplementary file 1 — Supplementary methods, figures and tables. [file ijbsv22p3322s1.pdf]

Supplementary Materials

(Methods, Figures, Tables)

Supplementary methods

Adipogenic Differentiation Induction and Oil Red O Staining

Adipogenic differentiation was performed using the Human MSC Adipogenic Induction and Staining Kit (Fuheng, Shanghai, Cat. No. WWL-G040) following the manufacturer's instructions. Briefly, MSCs were seeded at 2×10^5 cells per well in gelatin-coated 6-well plates with MSC-specific complete medium. Upon reaching 100% confluence, cells were induced with induction medium (Solution A) for 3 days alternating with maintenance medium (Solution B) for 1 day, for 3–5 cycles (12–20 days total), followed by maintenance with Solution B for 4–7 days. After induction, cells were fixed with 4% paraformaldehyde and stained with Oil Red O working solution (3:2 mixture of saturated stock solution and deionized water) for 30 min in the dark. Lipid droplet formation was observed under an inverted microscope.

Osteogenic Differentiation Induction and Alizarin Red Staining

Osteogenic differentiation was conducted using the Human MSC Osteogenic Induction and Staining Kit (Fuheng, Shanghai, Cat. No. WWL-G039) according to the manufacturer's protocols. MSCs were seeded at 1×10^5 cells/mL in gelatin-coated 6-well plates and cultured until 60%–70% confluence, then induced with osteogenic complete medium (replaced every 3 days). After 2–4 weeks of induction (when calcium nodules formed), medium replacement was adjusted to half-volume every 2 days. Cells were fixed with 4% paraformaldehyde and stained with Alizarin Red solution for 5–10 min. Calcium deposition was evaluated using an inverted microscope.

Chondrogenic Differentiation Induction and Alcian Blue Staining

Chondrogenic differentiation was carried out with the Human MSC Chondrogenic Induction and Staining Kit (Fuheng, Shanghai, Cat. No. WWL-G041) following the manufacturer's guidelines. A total of $3–4\times10^5$ MSCs were centrifuged in sterile 15 mL conical tubes to form cell pellets, which were cultured in chondrogenic induction medium (replaced every 2–3 days) for 21–28 days. Chondrospheres were fixed with 4% paraformaldehyde, dehydrated, embedded in paraffin, and sectioned at 4 μm thickness. Deparaffinized sections were stained with Alcian Blue working solution at 37°C for 1 h, and chondrogenic differentiation was observed under a microscope.

Mouse Hematological Collection and Serum Separation

At the experimental endpoint, mice were anesthetized using a small animal anesthesia machine, and blood was collected via orbital venous plexus puncture. Each mouse′ s blood sample was divided into two equal parts: one for serum separation and the other for hematological parameter detection. For serum separation, blood was allowed to coagulate at room temperature for 1 h, followed by centrifugation at 3000–4000 rpm at 4°C for 20 min. The upper serum was aspirated into a new EP tube and subjected to secondary centrifugation at 8000 rpm at 4°C for 5 min. The resulting supernatant was transferred to fresh EP tubes and stored at -20°C, avoiding repeated freeze-thaw cycles.

Drug Safety Assessment

During the administration period, changes in mouse body weight were monitored continuously. All mice were sacrificed simultaneously when tumor size reached the ethical standard. The collected blood was sent to Lanzhou Yurui Animal Hospital for blood cell counting and blood biochemical tests to evaluate the effects of the drug on the hematopoietic system, liver function, and renal function of mice. Meanwhile, organs including the heart, liver, spleen, lungs, kidneys, testes, and small intestine were collected for hematoxylin-eosin (HE) staining to assess whether the drug induced histopathological changes.

Supplementary Figures

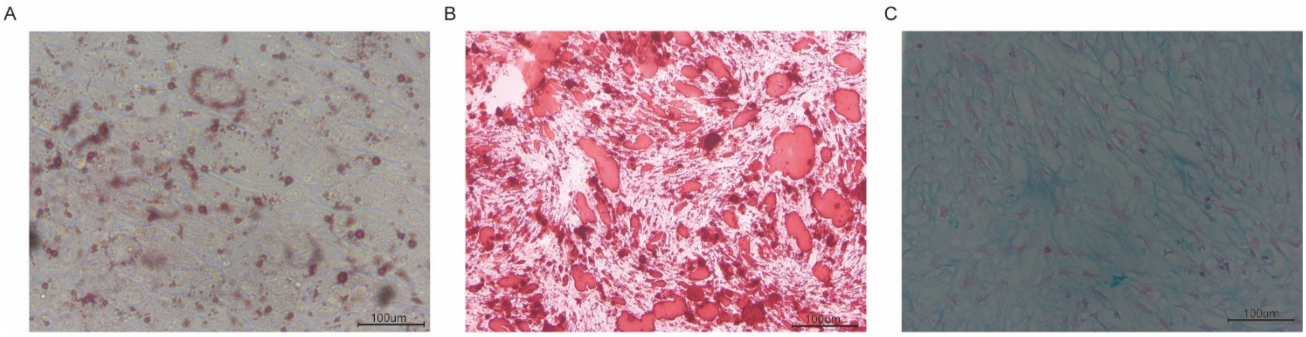

Fig.S1 MSCs exhibit adipogenic, osteogenic and chondrogenic differentiation potential
A, Oil Red O staining revealed numerous lipid droplets in MSCs at 15 days post adipogenic induction. **B,** Alizarin Red staining identified distinct red calcium nodules in MSCs following 21 days of osteogenic induction. **C,** Alcian Blue staining displayed abundant blue acid mucopolysaccharides in MSCs after 30 days of chondrogenic induction.

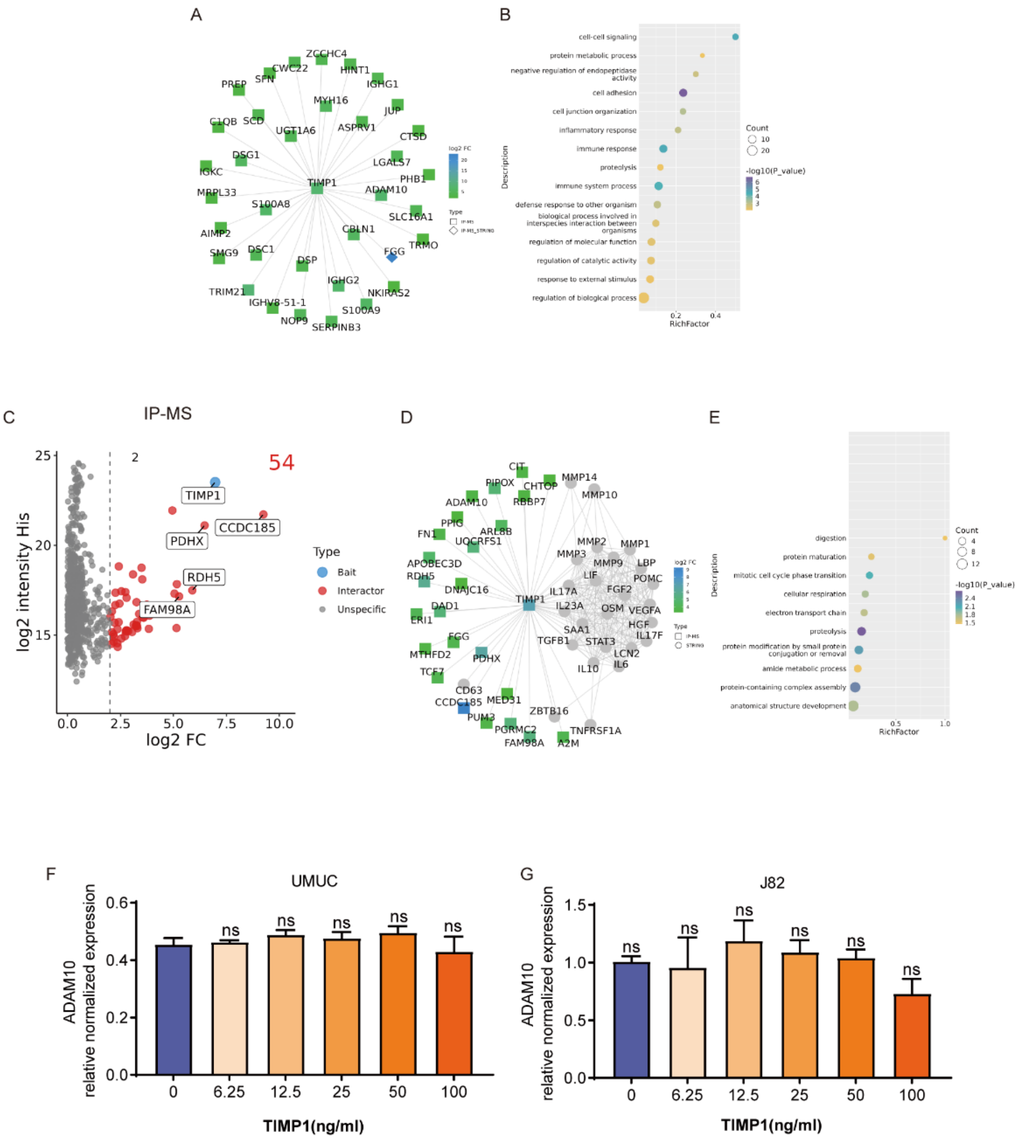

Fig.S2 The immunoprecipitation mass spectrometry detection results of TIMP1 interaction.

A, Network diagram between TIMP1 and the identified total proteins. **B,** Biofunctional enrichment analysis of total proteins interacting with TIMP1. **C,** Volcanic patterns of membrane proteins interacting with TIMP1 were identified. **D,** Network diagram between TIMP1 and the identified membrane proteins. **E,** Biofunctional enrichment analysis of membrane proteins interacting with TIMP1. **F-G,** ADAM10 qPCR results of UMUC-3 and J82 cells 24 hours after intervention with TIMP1 gradient concentration.

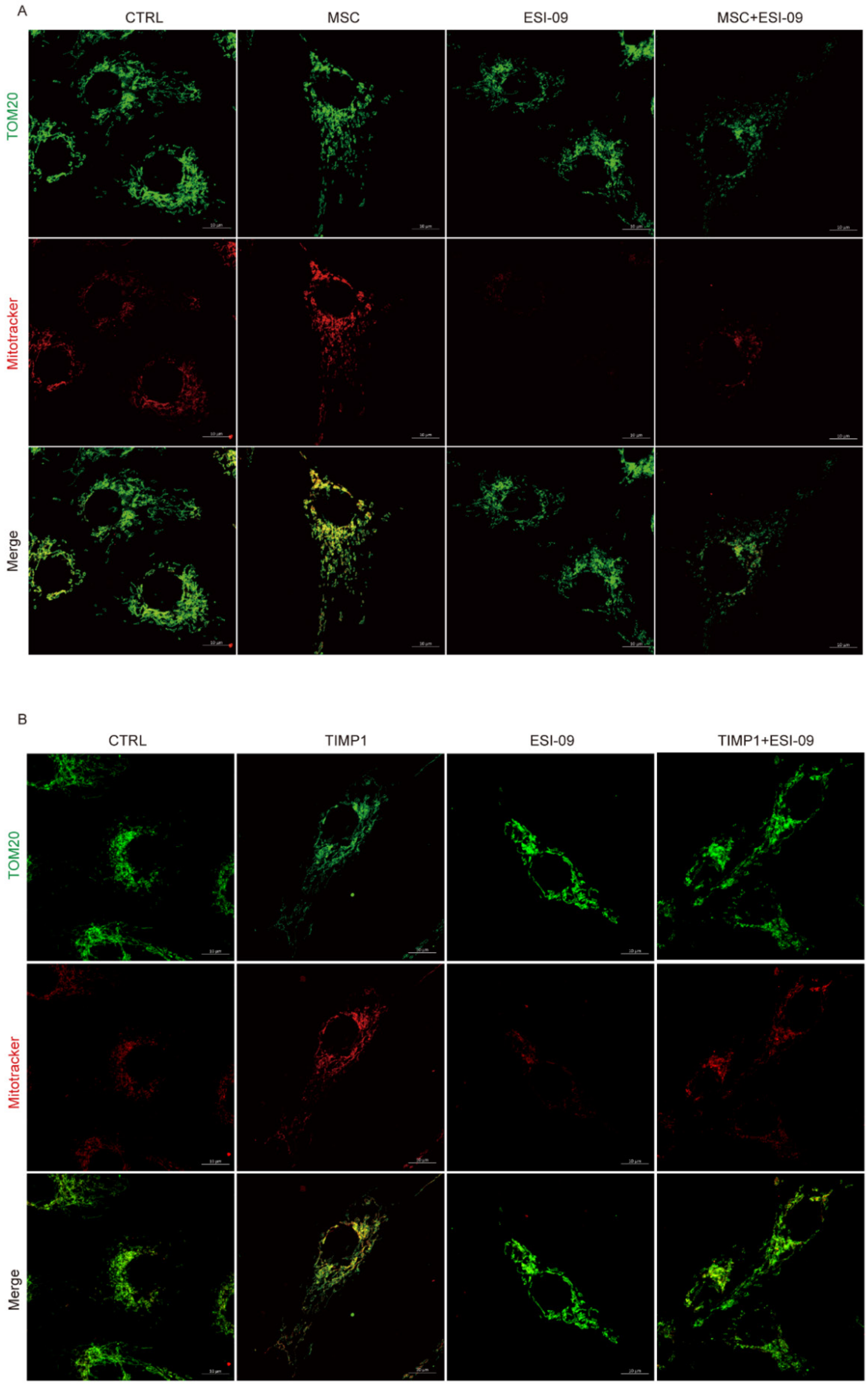

Fig.S3 Imaging of VDIMs markers following the intervention of UMUC-3 cells under different conditions.

A, VDIMs imaging of UMUC-3 cells 24 hours post-treatment with MSC-CM, ESI-09 10μM, and their combination.

B, VDIMs imaging of UMUC-3 cells 24 hours post-treatment withTIMP1 12.5ng/ml, ESI-09 10μM, and their combination.

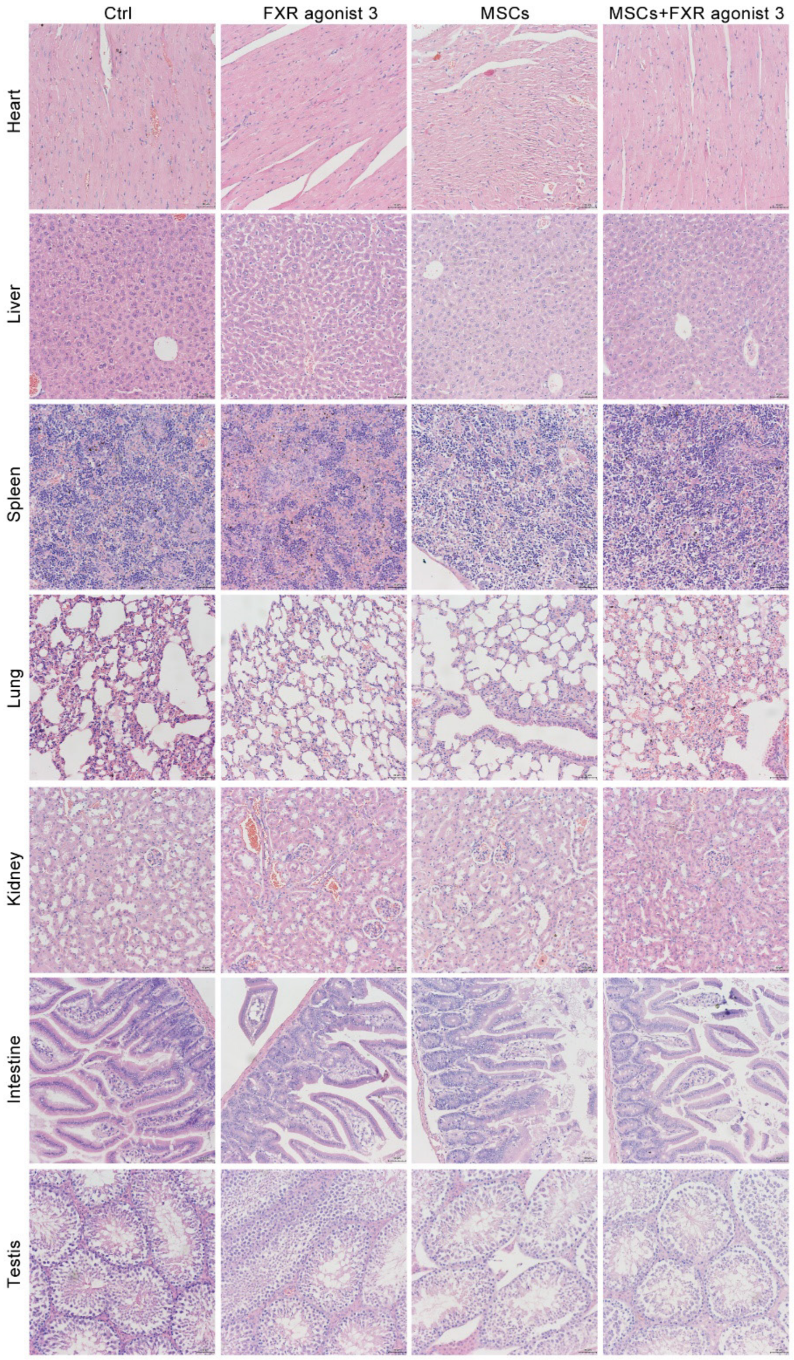

Fig.S4 Hematoxylin-eosin (HE) staining of the organs of mice in each group.

HE staining of organs such as the heart, liver, spleen, lung, kidney, testis and small intestine in mice of the CTRL group, FXR agonist 3 group, MSC group and MSC with FXR agonist 3 group.

Supplementary Tables

Table S1. Comparison of urinary TIMP1 levels (μg/ml) among bladder cancer patients with different clinical characteristics

Clinical feature	Sub group	n	TIMP1 (Mean±SD, μg/ml)	*P* value
Age (years)	<60 years	16	1.35±1.10	0.182
	≥60 years	26	2.71±3.02	
Gender	Female	3	1.07±0.66	0.379
	Male	39	2.28±2.61	
T stage	T1	28	1.20±1.04	0.000
	≥T2	14	4.17±3.43	
Pathological grade	low grade	14	1.16±1.35	0.006
	high grade	28	2.71±2.84	
Lesion type	Unifocal	30	1.85±2.14	0.122
	Multifocal	12	3.05±3.30	
Tumor history	Primary tumor	35	1.97±2.06	0.601
	Tumor recurrence	7	3.28±4.28	
Tumor volume (cm^3)	<1 cm^3	20	1.31±0.92	0.199
	≥1 cm^3	22	2.99±3.23	
KI67 index	<20	8	0.61±0.62	0.002
	≥20	32	2.64±2.74	
	Unknown	2	1.40±0.72	
p53 expression	Wild type	24	2.32±2.82	0.584
	Mutant type	10	1.65±1.71	
	Unknown	8	2.15±2.53	
C-erbB2 expression	0/1+	11	1.55±1.53	0.582
	2/3+	24	2.37±2.83	
	Unknown	7	2.02±2.27	

Notes: 1.All data were tested for normality using the Shapiro-Wilk test, and Mann-Whitney U test was applied for comparisons between two non-missing subgroups; 2.Tumor volume was calculated by the formula: $V=\pi/6\times L\times W\times H$.

Table S2. Hematological Indices of Mice in MSC Group and MSC + FXR agonist 3 Group

Abbreviation	Indicator	Unit	MSC Group (n=3)	MSC + FXR agonist 3 Group (n=3)	*P*-value
ALT	Alanine Aminotransferase	U/L	36 [33, 38]	30 [28, 33]	0.171
ALP	Alkaline Phosphatase	U/L	56 [52, 59]	43 [39, 45]	0.086
TBIL	Total Bilirubin	μmol/L	6.75 [6.05, 7.10]	7.41 [6.64, 7.81]	0.200
ALB	Albumin	g/L	35.6 [32.2, 38.8]	34.5 [30.4, 38.2]	0.857
TP	Total Protein	g/L	54.5 [51.04, 58.6]	53.2 [48.7, 56.4]	0.714
GLO	Globulin	g/L	18.9 [16.8, 20.4]	18.7 [17.0, 21.4]	0.943
AMY	Amylase	U/L	1154 [1078, 1287]	1075 [982, 1231]	0.686
CHOL	Cholesterol	mmol/L	3.5 [3.1, 3.9]	3.66 [3.2, 4.2]	0.771
GLU	Glucose	mmol/L	5.53 [4.86, 5.56]	4.29 [3.86, 4.60]	0.086
CRE	Creatinine	μmol/L	40 [36, 43]	31 [28, 36]	0.114
BUN	Blood Urea Nitrogen	mmol/L	6.76 [6.56, 6.88]	6.76 [6.56, 6.89]	0.971
CK	Creatine Kinase	U/L	1550 [1420, 1580]	1553 [1440, 1580]	0.857
Ca	Calcium	mmol/L	2.55 [2.23, 2.76]	2.61 [2.41, 2.80]	0.714
P	Inorganic Phosphorus	mmol/L	4.69 [4.21, 4.90]	5.15 [4.86, 5.40]	0.114
RBC	Red Blood Cell	10^{12}/L	9.13 [8.70, 9.60]	9.33 [8.70, 9.54]	0.857
WBC	White Blood Cell	10^{9}/L	4.2 [3.9, 4.5]	4.5 [3.9, 4.8]	0.686
PLT	Platelet	10^{9}/L	1302 [1180, 1490]	1910 [1860, 2010]	0.086
HGB	Hemoglobin	g/L	147 [140, 150]	148 [139, 152]	0.971

Note: 1. Data are presented as median [interquartile range]; 2. Statistical analysis was performed using the Mann-Whitney U test (independent samples nonparametric test);
